# Nano-Sized Iron Sulfide: Structure, Synthesis, Properties, and Biomedical Applications

**DOI:** 10.3389/fchem.2020.00818

**Published:** 2020-09-10

**Authors:** Ye Yuan, Liping Wang, Lizeng Gao

**Affiliations:** ^1^Key Laboratory for Molecular Enzymology and Engineering, The Ministry of Education, Jilin University, Changchun, China; ^2^School of Life Sciences, Jilin University, Changchun, China; ^3^CAS Engineering Laboratory for Nanozyme, Institute of Biophysics, Chinese Academy of Sciences, Beijing, China; ^4^Nanozyme Medical Center, School of Basic Medical Sciences, Zhengzhou University, Zhengzhou, China

**Keywords:** nano-sized iron sulfide, structure, synthesis, enzyme-like activities, biomedical applications

## Abstract

Nano-sized iron sulfides have attracted intense research interest due to the variety of their types, structures, and physicochemical properties. In particular, nano-sized iron sulfides exhibit enzyme-like activity by mimicking natural enzymes that depend on an iron-sulfur cluster as cofactor, extending their potential for applications in biomedicine. The present review principally summarizes the synthesis, properties and applications in biomedical fields, demonstrating that nano-sized iron sulfides have considerable potential for improving human health and quality of life.

## Introduction

With the development of nanotechnology (Li et al., 2019), nanomaterials have become a major resource for the development of novel therapeutic medicines and technologies designed to improve human health and the quality of life (Zhang and Webster, 2009; Esmaeili et al., 2020; Wang et al., 2020). In particular, due to their multiple functionality and excellent biocompatibility, iron-based nanomaterials are frequently used in the biomedical field, such as bioseparation, biosensors, magnetic resonance imaging (MRI), tumor hyperthermia, and drug delivery (Chen and Gu, 2017). In addition, recent studies have revealed that these nanomaterials have intrinsic enzyme-like properties (Gao et al., 2007; Xie et al., 2012; Xu et al., 2018), an important form of nanozyme representing a new generation of artificial enzyme (Wei and Wang, 2013; Dong et al., 2019; Liang and Yan, 2019). Currently, the majority of iron-based nanomaterials are iron oxide which possess excellent supraparamagnetic properties, with catalytic activity mimicking that of oxido-reductases, including peroxidase, catalase, superoxide dismutase, and oxidase (Gao et al., 2007; Liang and Yan, 2019). However, iron sulfide nanomaterials have not been comprehensively studied or used in the biomedical fields. Since O and S are congeneric elements, iron sulfide demonstrates similar physiochemical properties as iron oxide (Fu et al., 2019). In addition, the phases of iron sulfide in nature include mackinawite (FeS), pyrrhotite (Fe_1−x_S), pyrite (FeS_2_), and greigite (Fe_3_S_4_), etc., which exhibit more variability than iron oxide containing only Fe_2_O_3_ and Fe_3_O_4_. The band gap in iron sulfide is smaller than that of iron oxide, leading to the former having more appropriate electron transfer and conductivity (Wadia et al., 2009; Jin et al., 2017; Zhang et al., 2018). Importantly, iron-sulfur clusters are important cofactors in many enzymes which serve as active centers for electron transfer in catalytic processes and respiratory chain reactions (Qi and Cowan, 2011). Therefore, it is anticipated that iron sulfide nanomaterials will display multiple functionalities and they have great potential in biomedical applications. Herein, we will summarize the types, synthesis and properties of iron sulfide nanomaterials and emphasize their applications in biomedical and medical fields. This will provide a comprehensive understanding of iron sulfide nanomaterials and illustrate their considerable potential as novel multifunctional biomaterials in biomedical applications.

## Type and Structure of Iron Sulfide

Solid phases of iron sulfides principally comprise FeS (mackinawite), Fe_1−x_S (pyrrhotite), FeS_2p_ (pyrite), FeS_2m_ (marcasite), Fe_3_S_4_ (greigite), and Fe_9_S_11_ (smythite). The content of iron within a biomaterial therefore influences its phase, shape, and physical and chemical properties. FeS naturally has a tetragonal structure, with each iron atom coordinated to four sulfurs. For Fe_1−x_S, a monoclinic hexagonal is present. FeS_2p_ forms stable iron (II) disulfides with cubic structures. FeS_2m_ differs from FeS_2p_ as an orthorhombic metastable iron (II) disulfide, whilst Fe_3_S_4_ is a cubic metastable Fe (II) Fe (III) sulfide. Hexagonal Fe_9_S_11_ is related to the Fe_1−x_S phase (Rickard and Luther, 2007).

**Figure 1:**
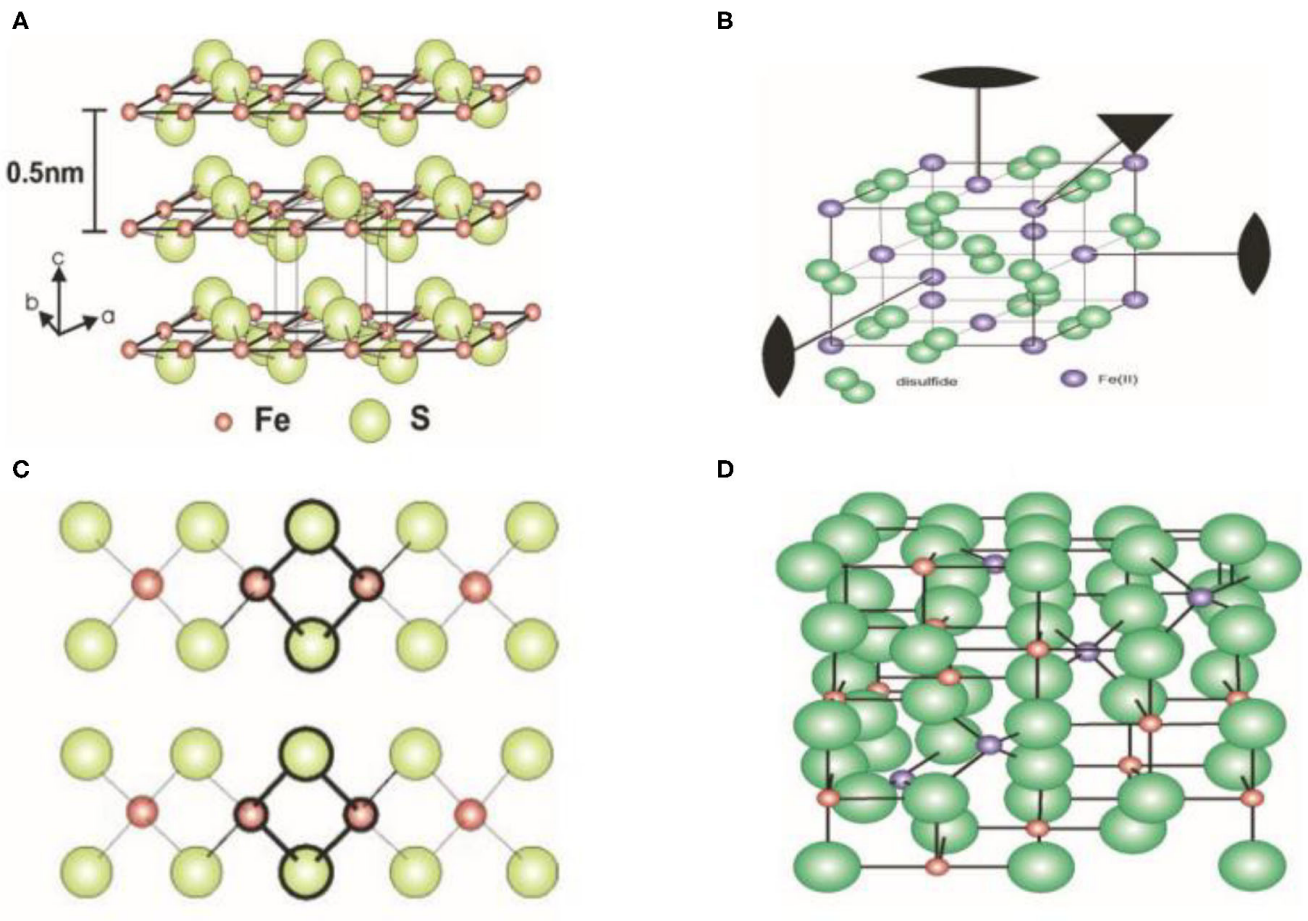
Crystal structure of iron sulfide. **(A)** FeS. **(B)** FeS_2_. **(C)** Fe_2_S_2_. **(D)** Fe_3_S_4_. Reproduced with permission from Rickard and Luther (2007). Copyright 2007, American Chemical Society.

Reported crystal structures of iron sulfide are displayed in Figure 1 (Fleet, 1971; Argueta-Figueroa et al., 2017). FeS possesses a tetragonal layered structure in which the iron atoms are linked through tetrahedral coordination to four equidistant sulfur atoms. A single iron atom is coordinated to four equidistant sulfur atoms. The distance of Fe-Fe is 2.5967 Å. In addition, Fe-Fe bonding is substantial in FeS. To assess the effects of van der Waals forces resulting from the S atoms, sheets including Fe are stacked along the C-axis. The spacing of these layers is 5 Å. The structure of Fe_2_S_2_ is closed to FeS. The structure of FeS_2_ is similar to that of NaCl in which S^2−^ is located at the center of a cube. The cubic structure has a low symmetry. In addition, FeS_2_ exhibits chirality through absorbed organic molecules. Fe_3_S_4_ has an inverse spinel structure in which 8 Fe atoms are located at the tetrahedral A-sites and 16 Fe atoms are located at the B-sites of the octahedron. The unit cell of Fe_3_S_4_ is 9.876 Å. In addition, the cubic structure of Fe_3_S_4_ forms a closely packed array of S molecules linked by smaller Fe units (Figure 1D). It has been established that the Fe_7_S_8_ structure is a hexagonal supercell (Fleet, 1971). The viable distribution of vacancy sites ideal for the base structure of NiAs was observed to describe the structure of Fe_9_S_10_ (Elliot, 2010).

## Synthesis of Nano-Sized Iron Sulfides

Nano-sized iron sulfide encompasses a range of iron and sulfur compounds. Firstly, the range of chemical and biological methods for their production are discussed. In addition, the most reported synthesized methods for creating different phases of iron sulfide are presented in Table 1.

**Table 1:** Appearances, sizes and lattice spaces of iron sulfide.

**Phase**	**Method**	**Appearances**	**Size (nm)**	**Lattice space (nm)**	**References**
FeS	High temperature chemical synthesis	Nanoplates	32–36	0.286	Yang et al., 2015
	Sonochemical synthesis	Spherical	6–20		Ahuja et al., 2019
	Biosystem	Spherical	30–50		Mei and Ma, 2013
	Hydrothermal synthesis	Pomegranateflower-like	3,000		Jin et al., 2017
	Co-precipitation Co-precipitation	Regular sphericalNanoparticles	50 60–80 102 (DLS)		Dai et al., 2009; Agnihotri et al., 2020
	Biomineralization	Spherical	2		Watson et al., 1999
FeS_2_	High temperature chemical synthesis	Cubic	60–200		Bi et al., 2011
	Ionic liquid-modulated synthesis	Hexagonal nanoplates	12,000 30 (side length)		Ma et al., 2010
	Hydrothermal synthesis	Quasi-cubic nanocrystal	Over 100		Wadia et al., 2009
	Microwave	Big particles	200		Kim and Batchelor, 2009
	Hydrothermal synthesis	Uniform nanowires	40–60	0.269	Kar and Chaudhuri, 2004
	Hydrothermal synthesis	Nanorods	40–100		Kar and Chaudhuri, 2004
	Hydrothermal synthesis	Nanoribbons	100–250	0.313	Kar and Chaudhuri, 2004
	Microwave	Monodisperse microspherolites	2,400	0.27	Li M. et al., 2011
	Biomineralization	Nanodots	7		Jin et al., 2018
	Low temperature synthesis	Hexagonal	600–700		Srivastava et al., 2014b
Fe_3_S_4_	Hydrothermal synthesis	Spherical	76 165 (DLS)		Ding et al., 2016
	Hydrothermal synthesis Hydrothermal synthesis	PlatesDispersible nanoparticles	2000–5000 17.7	0.298	Fu et al., 2019; Moore et al., 2019
	Hydrothermal synthesis Hydrothermal synthesis Hydrothermal synthesis	NanocrystalsHexagonal nanolpatesMonodisperse nanocrystals	2.5–4.5 12,000 30 (side length) 100 175 (DLS)		Vanitha and O'Brien, 2008; Ma et al., 2010; He et al., 2013
	Co-precipitation Co-precipitation Co-precipitation	Irregular particlesNanoparticlesPlatelet-like	50–100 20–35 10–20	0.572	Chang et al., 2011; Paolella et al., 2011; Simeonidis et al., 2016
	High temperature chemical synthesis	Spherical	5	0.25	Beal et al., 2012
Fe_7_S_8_	Hydrothermal synthesis	Spherical	5.6		Vanitha and O'Brien, 2008
	Flux	hedgehog-like	10,000		Kong et al., 2005
	Flux	Nanorods	200 1500–2000 (length)		Kong et al., 2005
	Flux	Nanosheets	Smaller than 100 (thickness)		Kong et al., 2005
	Hydrothermal synthesis	Nanowires		0.289	Yao et al., 2013
	High temperature chemical synthesis	Nanosheets	5,000 500 (thickness)		Wang et al., 2013

### Hydrothermal Synthesis

Thermal decomposition is the most commonly-used hydrothermal reaction for iron sulfide production. The typical solvothermal synthesis method of nFeS firstly involves the dissolution of FeCl_3_·6H_2_O in 40 mL of ethylene glycol. NaOAc and organosulfur compounds (allyl methyl sulfide, diallyl sulfide, diallyl trisulfide, diallyl disulfide, cysteine, cystine, glutathione (GSH), or methionine) are then added under continuous and vigorous stirring. The system was then sonicated for 10 min and transferred to a Teflon-lined stainless steel autoclave. The mixture was reacted at 200°C for 12 h and precipitates washed three times with ethanol and water. Finally, the products were dried at 60°C for 3 h (Xu et al., 2018). For FeS_2_, the single source molecular precursor Fe^3+^ diethyl dithiophosphate forms an aqueous solution through the reaction of FeCl_3_ and (C_2_H_5_O)_2_P(S)SNH_4_, with hexadecyltrimethylammonium bromide (CTAB) added as a surfactant and reacted with a single precursor [(C_2_H_5_O)_2_P(S)S]_3_Fe (Wadia et al., 2009). For FeS, FeCl_3_·6H_2_O was dissolved in ultrapure water with ethanolamine and thiourea added to the solution. After stirring for 25 min, the mixture was added to a Teflon lined autoclave and reacted at 180°C for 12 h. The synthesis of Fe_3_S_4_ differs from that of FeS. FeCl_3_·6H_2_O, ethylene glycol, thiourea and H_2_O_2_ were mixed and reacted at 180°C for 18 h in the presence of the capping agent polyvinyl pyrrolidone (PVP) to prevent excessive growth and aggregation of the nanoparticles (NPs) (Moore et al., 2019). Fe_1−x_S single crystals were then synthesized through a hydrothermal method by the addition of K_0.8_Fe_1.6_S_2_ crystals, Fe powder, NaOH, and thiourea in deionized water, which was reacted at 120°C for 3–4 days (Guo et al., 2017). Ionic liquids that form extended hydrogen bond systems were then used to form higher structures in the base of the hydrothermal process, as reported by Zheng and colleagues when changing the structure of Fe_3_S_4_ (Ma et al., 2010). Generally speaking, the product obtained by the hydrothermal method has better dispersibility and controllability, but iron oxide impurities can also appear during the synthesis of iron sulfide. Meanwhile, multiphase iron sulfide appears to occur easily, as assessed by X-ray diffraction (XRD) patterns for hydrothermally-synthesized samples.

### Microwave Production

The principal advantages of microwave-assisted methods, compared with conventional heating include its reduced reaction time, smaller particle size distribution, and higher purity. Ethylene glycol is a solvent suitable for microwave-assisted methods owing to its relatively high dipole moment. For FeS_2_ microspherolites, FeSO_4_·7H_2_O, PVP-K30 and S powder in ethylene glycol can be reacted by microwave in an N_2_ atmosphere (Li M. et al., 2011). Although this emerging technique may be more desirable it should be noted that the aggregation phenomenon does not appear to be improved.

### Co-precipitation

Chemical co-precipitation does not introduce impurities. The operation is performed under mild conditions and is typically synthesized using Fe_3_S_4_ methods, in which iron (II) sulfate heptahydrate and sodium sulfide are dissolved in ultrapure deionized water. The solution was then added dropwise to acetic acid to adjust the pH to 3.0, followed by stirring for several minutes. The reaction was prepared under an N_2_ atmosphere (Chang et al., 2011). In addition, green synthesis was achieved in a continuous stirred-tank reactor (Simeonidis et al., 2016). As previously reported, the conditions of synthesis required by co-precipitation are harsher than those of other methods, and the products obtained may show poor homogeneity.

### High Temperature Chemical Synthesis

Chemical synthesis methods using high temperatures have been reported for FeS_2_. Briefly, iron (II) acetylacetonate (Fe(acac)_2_), trioctylphosphine oxide (TOPO) and oleyamine (OLA) were mixed and degassed at 110°C for 1 h under a vacuum. The mixture was then rapidly heated to 220°C for 1 h under vigorous magnetic stirring in the presence of nitrogen. Sulfur was then quickly injected into the solution, which was heated to 220°C for 1 h. Once the solution cooled, ethanol was added to the precipitate to develop the FeS_2_ nanoplates (Bi et al., 2011). Synthesis methods for Fe_1−x_S and Fe_3_S_4_ have also been reported. The rapid injection method has been used to reduce the size of Fe_3_S_4_ (Beal et al., 2012). This method of synthesis is highly sensitive to the experimental conditions.

### Sonochemical Synthesis

As a convenient and stable synthetic method, Bala and colleagues described specific FeS' sonochemical synthesis. Firstly, sodium sulfide was dissolved in double distilled water. FeSO_4_·7H_2_O was then independently dissolved in a solution of double distilled water and polyethylene glycol (1:1). The sodium sulfide solution contained a drop of Triton-X surfactant which was added dropwise to the above solution while being continuously sonicated for 30 min. PVP was then added and the system was mixed via ultrasound for a further 30 min (Ahuja et al., 2019).

### Other Chemical Methods

Some unusual synthetic chemical methods have been reported. The low temperature synthesis of FeS_2_ nanoparticles was described in 2014 (Srivastava et al., 2014a). Briefly, FeCl_3_ and sodium polysulfide (Na_2_S_x_) were mixed in pH 5.6 acetate buffer in an anaerobic environment. The black solution then was reacted in a 90–100°C oil bath for 4 h to produce a grayish FeS_2_ product. Flux methods were then used to synthesize 1D Fe_7_S_8_. The reaction was conducted within a furnace at 750–850°C (Kong et al., 2005). FeS can also be created in a biological system (Mei and Ma, 2013).

### Biomineralization

The bio-synthesis of iron sulfide using microorganisms is superior for biomedical applications (Li X. et al., 2011). When microorganisms interact with target ions, they are transported into microbial cells to form NPs in the presence of specific enzymes. In addition to the advantages of green synthesis, biological methods improve the biocompatibility of iron sulfide. The particles generated have higher catalytic reactivity and a greater surface area. Previous studies have reported that FeS_2_, Fe_3_S_4_, and FeS NPs can be produced by microorganisms. For the biomineralization of Fe_3_S_4_ and FeS_2_, a magnetotactic bacterium has been described (Mann et al., 1990). In 1995, FeS materials were produced by sulfate-reducing bacteria grown on iron containing substrates (Watson et al., 1995). Bazylinski and colleagues also reported the formation of Fe_3_S_4_ using non-cultured magnetotactic bacteria (Lefèvre et al., 2010). Sulfate-reducing bacteria were able to produce Fe_1−x_S as reported by Charnock and colleagues (Watson et al., 2000). NPs were subsequently formed at the surface by the microorganisms and as such, the porous structure of the iron sulfide NPs failed to prevent normal metabolism. These studies verified the utility of this method for efficient NP production. Chemical biomineralization methods have also been used to synthesize FeS_2_ and FeS Quantum dots (QDs) (Jin et al., 2018; Yang et al., 2020).

### Iron Sulfide Modifications

Bare nanocrystal cores have an unstable structure that is prone to photochemical degradation. However, those that are unmodified display higher toxicity. As such, biocompatible moieties are essential as they serve as caps for nanomaterials, including polyethylene glycol (PEG), silica, lactose, citrate, and dextran (Simeonidis et al., 2016; Mofokeng et al., 2017). Previous studies have reported the synthesis, characterization, cytotoxicity and biodistribution of FeS/PEG nanoplates *in vivo* (Yang et al., 2015). An adsorption-reduction method was used to load silver onto the surface of 3-aminopropyltriethoxysilanemodified 3-aminopropyl triethoxysilane (APTES)-modified Fe_3_S_4_ particles, achieving the preparation of magnetic composite nanoparticles of Fe_3_S_4_/Ag (He et al., 2013). However, modifications also led to adverse effects. For example, the modification of CTAB inhibited the growth of nano Fe_3_S_4_ (Simeonidis et al., 2016).

### Characterization of Iron Sulfide

**Figure 2:**
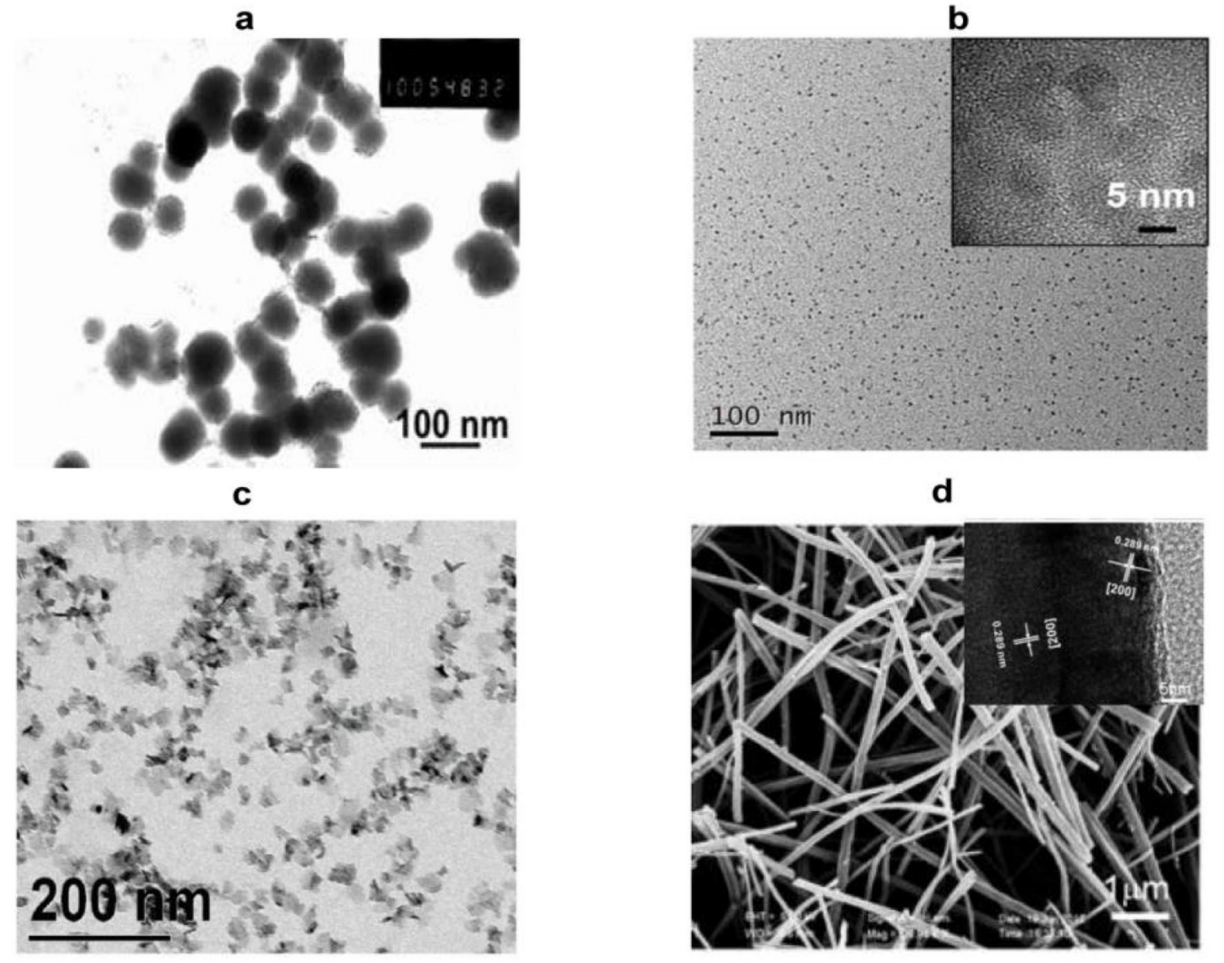
Representative images of nano-sized iron sulfide. **(A)** TEM image of the spherical FeS. Reproduced with permission from Dai et al. (2009). Copyright 2009, Wiley-VCH Verlag GmbH & Co. KGaA, Weinheim. **(B)** TEM and HRTEM of FeS_2_ nanodots. Reproduced with permission from Jin et al. (2018). Copyright 2017, American Chemical Society. **(C)** TEM images of platelet-like Fe_3_S_4_. Reproduced with permission from Paolella et al. (2011). Copyright 2011, American Chemical Society. **(D)** SEM and HRTEM of Fe_7_S_8_ nanowires. Reproduced with permission from Yao et al. (2013). Copyright 2013, Wiley-VCH Verlag GmbH & Co. KGaA, Weinheim.

Detailed characterization is required to confirm the synthesis of iron sulfide. Scanning electron microscopy (SEM) and transmission electron microscopy (TEM) can be used to image surface morphology. These represent the most direct and commonly-used methods to assess the microstructure, size, and dispersion of the materials. Differences in the synthetic methods have presented variations in TEM/SEM imaging, including within the same phase. For FeS, nanoplates, spherical nanoparticles, and pomegranate flower-like shapes have been reported. FeS_2_ exists as large particles, cubic nanocrystals, monodisperse microspherolites, spherical and hexagonal nanoribbons, and nanorods. Typical images of iron sulfide are shown in Figure 2. Table 1 reports the most common morphology and size of FeS, FeS_2_, Fe_3_S_4_, and Fe_7_S_8_. Lattice spaces are measured to judge the crystallinity of the materials. The reported lattice spaces of the iron sulfides are summarized in Table 1. The composition, content, and structure of a substance can be analyzed, measured, and inferred using Ultraviolet-visible (UV-Vis) spectrometry and the absorption of UV and visible light. According to previous studies, the UV absorption peaks of FeS are 285 nm and 500 nm (Table 2). Information on the composition of the materials and the structure and/or morphology of the atoms or molecules inside the materials can be obtained by XRD analysis. XRD is the most direct indicator of whether a crystal is pure and so represents a convenient system to analyze synthesized materials. JCPDS cards are used to contrast and analyze unknown crystals. JCPDS cards of FeS (JCPDS card No. 15-0037 and 75-0602), FeS_2_ (JCPDS card No. 03-065-1211, 89-3057, 65-3321, and 42-130), Fe_3_S_4_ (JCPDS card No. 16-0713, 89-1998, and 16-0073) and Fe_7_S_8_ (JCPDS card No. 25-0411 and 76-2308) are listed in Table 2. For iron sulfide nanomaterials, the X-ray photoelectron spectroscopy (XPS) of Fe and S are essential. FeS materials are presented in Table 2 (Fe 2p: 2p_3/2_ (711.4 eV), 2p_1/2_ (724.9 eV), S 2p: 2p_3/2_ (161.1 eV), 2p_1/2_ (166.0 eV)), FeS_2_ (Fe 2p: 2p_3/2_ (707.0 eV), 2p_1/2_ (720.0 eV), S 2p 2p_3/2_ (162.3 eV), 2p_1/2_ (163.5 eV)), Fe_3_S_4_ (Fe 2p: 2p_3/2_ (711.0 eV), S 2p: 2p_3/2_ (161.0 eV), 2p_1/2_ (162.5 eV)), Fe_7_S_8_ (Fe 2p: 2p_3/2_ (709.9 eV), (711.6 eV), S 2p: 2p_3/2_ (163.5 eV), 2p_1/2_ (164.7 eV)). Fourier transform infrared (FTIR) spectroscopy can be used to detect functional groups in a complex mixture and so is therefore essential to the characterization of modified iron sulfide. Previous studies have provided FTIR spectra, showing successful modifications by APTES on the surface of the Fe_3_S_4_ nanoparticles (He et al., 2013). Fe_7_S_8_/N-C nanohybrids were prepared for FeMOF and FeMOF-S and analyzed by FTIR (Jin et al., 2019). In addition, energy dispersive spectroscopy (EDS) (Paolella et al., 2011; Ding et al., 2016; Guo et al., 2016), dynamic light scattering (DLS) (He et al., 2013; Ding et al., 2016), Raman spectroscopy (Gan et al., 2016; Guo et al., 2016), selected area electron diffraction (SAED) (Kar and Chaudhuri, 2004), X-ray absorption fine structure (XAFS) (Feng et al., 2013), differential scanning calorimetry (DSC) and thermogravimetric analysis (TGA) (Jin et al., 2019), nitrogen adsorption–desorption isotherms and pore size distribution (Guo et al., 2016) analysis of iron sulfide have been reported as characterization systems.

**Table 2:** UV-VIS, XPS and XRD of iron sulfide.

**Phase**	**UV-Vis peak (nm)**	**XPS**	**XRD**	**References**
FeS	500 285 A broad absorption in (400–700 nm)	Fe 2p 2p_3/2_ (711.4 eV) 2p_1/2_ (724.9 eV)S 2p 2p_3/2_ (161.1 eV) 2p_1/2_ (166.0 eV)	JCPDS card No.15-0037 JCPDS card No.75-0602	Guo et al., 2016; Jin et al., 2017; Ahuja et al., 2019; Agnihotri et al., 2020
FeS_2_		Fe 2p 2p_3/2_ (707.0 eV) 2p_1/2_ (720.0 eV)S 2p 2p_3/2_ (162.3 eV) 2p_1/2_ (163.5 eV)	JCPDS card No.03-065-1211 JCPDS card No.89-3057 JCPDS card No.65-3321 JCPDS card No.42-1340	Kar and Chaudhuri, 2004; Wadia et al., 2009; Li M. et al., 2011; Gan et al., 2016
Fe_3_S_4_		Fe 2p 2p_3/2_ (711.0 eV)S 2p 2p_3/2_ (161.0 eV) 2p_1/2_ (162.5 eV)	JCPDS card No.16-0713 JCPDS card No.89-1998 JCPDS card No.16-0073	Ma et al., 2010; Chang et al., 2011; Paolella et al., 2011; Beal et al., 2012; Feng et al., 2013; He et al., 2013; Ding et al., 2016; Moore et al., 2019
Fe_7_S_8_		Fe 2p 2p3/2 (709.9 eV) (711.6 eV)S 2p 2p_3/2_ (163.5 eV) 2p_1/2_ (164.7 eV)	JCPDS card No. 25-0411 JCPDS card No.76-2308 JCPDS card No.71-0647	Kong et al., 2005; Vanitha and O'Brien, 2008; Wang et al., 2013; Yao et al., 2013; Jin et al., 2019

## Characteristics of Nano-Sized Iron Sulfides

In addition to both the physical and chemical properties, characteristics include the structure, solubility, stability, reactivity, magnetic properties, and photothermal properties of the products.

### Solubility

The major forms of nano-sized iron sulfides are solid precipitates which have poor solubility in water. However, Rickard et al. revealed that sedimental FeS can dissolve at c (S^2−^) ≤ 10^−5.7^ M to form Fe^2+^. The solubility can be increased in an alkaline as opposed to neutral environment (Rickard, 2006). FeS does not dissolve in HCl, meaning it cannot be removed with HCl (Rickard and Luther, 2007). According to previous studies, the K_sp_ of FeS_2_ was 10^−16.4^ at 25°C. The solubility products of various iron sulfides were assessed and resulted in consensus values for the pKs (FeS: 3.6 ± 0.2; FeS_2_ 16.4 ± 1.2; Fe_3_S_4_ 4.4 ± 0.1; Fe_7_S_8_ 5.1 ± 0.1). This improved our understanding of the solubility of iron sulfide in both synthetic and natural water at room temperature (Davison, 1991).

### Stability

FeS is stable within the P-T range of the Martian core (Kavner et al., 2001). Understanding the relationship between the stability of iron sulfide and its chemical environment is of key importance. Once iron sulfide is formed, its structure is reversible. Studies have examined the stability of FeS_2_ in different temperatures and the concentrations of absorbed water on the surface. Temperature has little effect on the morphology of FeS_2_ under low absorbed water concentrations. However, at above 90 K, the conversion from an octahedral structure to a cubic shape is promoted. At higher concentrations of water, the dependence on temperature is more apparent (Barnard and Russo, 2009). The latter study established that functions of the surface ligands affect the stability of FeS_2_ (FeS_2_ nanorods synthesized in laboratory) (Barand and Russo, 2009). The stability of FeS contributed to low energy excitation from Fe d to S-Sσ^*^p (Zhang et al., 2018). Fe_3_S_4_ was observed at 200°C for 30 h then it transformed to FeS_2_ over time (Gao et al., 2015).

### Reactivity

Iron sulfide is highly reactive to N_2_ and H_2_S. This reaction occurs at room temperature and the adsorption of N_2_ is dependent on the surface FeS and on the electronic state of N_2_. A decrease in absorbed N_2_ and H_2_S could be explained by the formation of ammonia (Kasting, 1993). The reactivity of Fe_7_S_8_ is similar to FeS (Niño et al., 2018). The presence of both Au^1+^ and Au atoms has been observed on the surface of FeS_2_. Au deposition increased at higher pH and temperatures. The reactivity of Au^1+^ sulfides with FeS_2_ have also been investigated (Scaini et al., 1998). The reactivity of FeS_2_ using gaseous H_2_O and O^2−^ was similarly reported. Gaseous H_2_O leads to the formation of iron hydroxides on FeS_2_. A sequence of different exposures also leads to the formation of a range of products (${\text{SO}}_{4}^{2-}$, Fe(OH)_3_) (Usher et al., 2005). Recently, FeS_2_ was shown to be a potential nanomaterial for prebiotic chemistry due to its highly reactive surface that drives amino acid adsorption (Ganbaatar et al., 2016). Among the most common probes, water molecules have been used to explore the reactivity of FeS_2_ (De Leeuw et al., 2000). The different phases of iron sulfide display a wide range of reactivities to chlorinated solvents. Conditions including pH, sulfide concentrations, metal ions, and natural organic matter can affect the reaction kinetics of the degradation of chlorinated solvents (He et al., 2015). The interaction of FeS, Fe_3_S_4_ and CO_2_ have also been reported. The charge transfer on FeS can also effectively activate CO_2_, whilst Fe_3_S_4_ is unreactive to CO_2_ (SantosCarballal et al., 2017).

### Magnetic Properties

**Figure 3:**
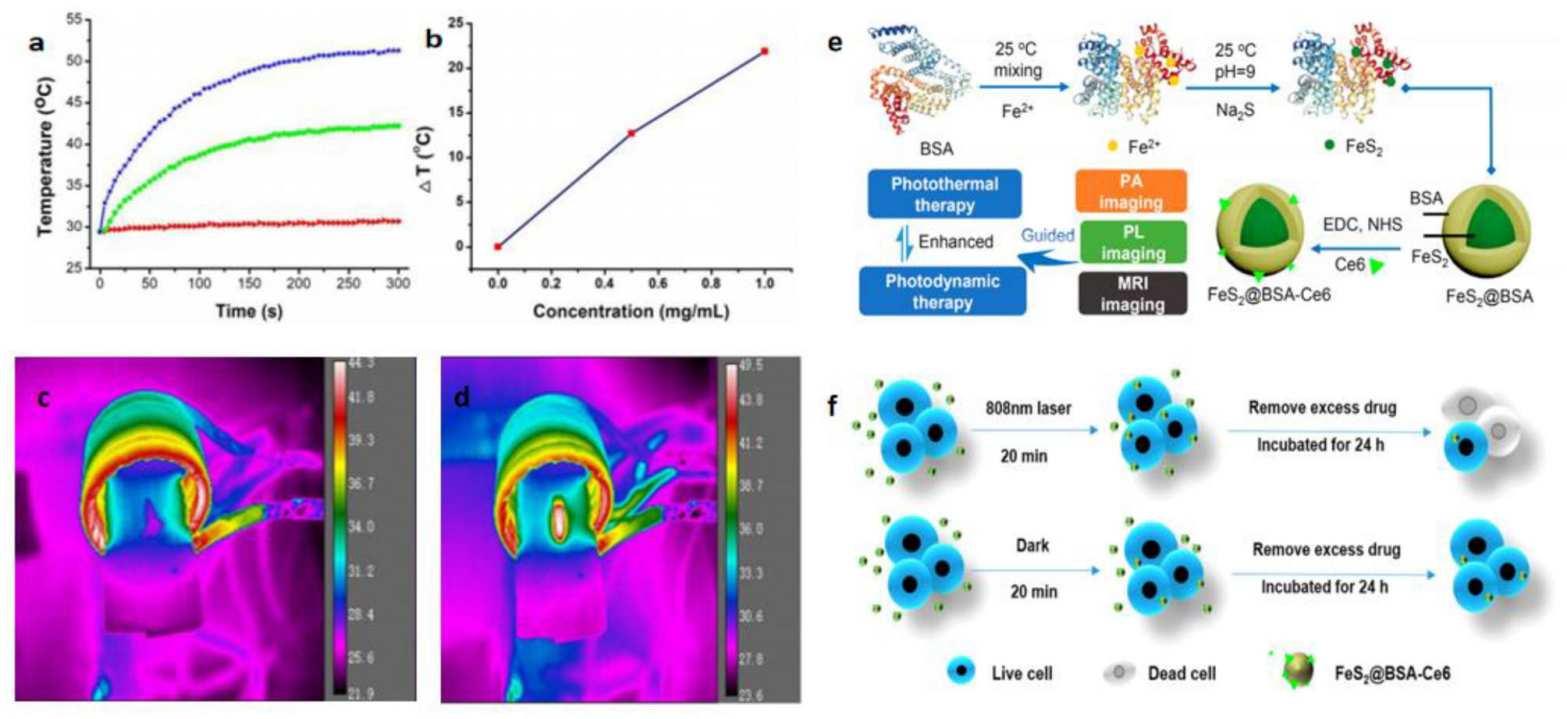
Magnetocaloric conversion performance **(a–d)** and photothermal property of iron sulfides. **(a)** Temperature change of the Fe_3_S_4_ nanoparticles in water at varied concentrations of Fe^2+^ (i.e., 0, 0.5, and 1.0 mg/mL) as a function of magnetic field action time. **(b)** Plot of temperature change over 300s vs. the concentration of Fe_3_S_4_ nanoparticles. Thermal imaging of **(c)** pure water and **(d)** Fe_3_S_4_ nanoparticles (1.0 mg/mL) under the action of an AMF for 5 min. Reproduced with permission from Fu et al. (2019). Copyright 2019, Frontiers in Materials. **(e)** Synthetic route and applications of FeS_2_ nanodots in the base of its photothermal activity. **(f)** Scheme showing photothermal enhanced cellular uptake of FeS_2_@BSA-Ce6 nanoparticles. Reproduced with permission from Jin et al. (2018). Copyright 2017, American Chemical Society.

Nanomaterials with magnetic properties have numerous applications, including magnetocaloric therapies, as MRI agents, magnetic separation materials, and magnetic carriers. The discovery of their magnetic properties led to the identification of iron sulfide phases. The ferromagnetism of Fe_7_S_8_ can be explained by Fe^3+^ ions with excess sulfur (Yosida, 1951). The magnetic susceptibility χ of natural FeS_2_ was found to be 64 × 10^−6^68 × 10^−6^ cm^3^ /moles between 4.2 and 380 K (Mohindar and Jagadeesh, 1979). Magnetic ordering in FeS was inferred and used to prove strong itinerant spin fluctuations. FeS can also be used as a superconductor (Kwon et al., 2011). Even when the structure of FeS is changed from troilite to the MnP-type under high pressure, the antiferromagnetic properties are preserved until the monoclinic structure is formed (Ono, 2007). The magnetic moment then disappears and yetragonal-phase FeS (Tc: 5K) was observed for the same structure as the superconductor FeSe (Tc: 8 K) (Kuhn et al., 2016). Fe_3_S_4_ displays high Mrs/χ, (Mrs/χ: the saturation isothermal remnant magnetization: magnetic susceptibility) and its MrsMs (hysteresis ratios) and Bcr/Bc are 0.5 and 1.5 (Ms: saturation magnetization; Bc: the coercive force; Bcr: the coercivity of remanence). Fe_3_S_4_ also displays unique high-temperature properties, with a clear drop in magnetization from 270 to 350°C (Roberts, 1995). Synthesized Fe_3_S_4_ contains various crystals from small superparamagnetic grains (non-remanence) to large multi-domain grains (Snowball, 1991). The magnetic hysteresis properties of Fe_7_S_8_ have also been studied (Menyeh and O'Reilly, 1997). The relationship between structure and magnetic properties has been reported within variable temperatures. Magnetic transitions occurred within the transformation of the structure (Powell et al., 2004). The magnetocaloric conversion ability of Fe_3_S_4_ nanoparticles has been measured under an alternating magnetic field (AMF). Meanwhile, the excellent physical and chemical properties provide magnetothermal thrombolytic ability in medical applications (Fu et al., 2019; Figure 3).

### Photothermal Properties

Photothermal therapy (PTT) has attracted considerable attention in recent years. The mechanism of PTT results mainly from photo-absorbing nanomaterials that generate heat through continuous laser irradiation, destroying cancer cells, but causing no damage to healthy tissue. It is therefore necessary to pay attention to the photothermal properties of iron sulfide. PEGylated FeS (FeS-PEG) nanoplates exhibit high near infrared (NIR) absorbance. Using Infrared (IR) thermal imaging, the temperature can reach 70°C within 5 min. Meanwhile, FeS-PEG displays stronger photothermal conversion efficiency than other known iron oxides (Yang et al., 2015). Ultrasmall FeS_2_ nanodots have also been synthesized and have been shown to be useful for photodynamic therapy. Chlorin e6 (Ce6) was used to conjugate FeS_2_ in the presence of the template bovine serum albumin (BSA). FeS_2_@BSA-Ce6 nanodots (7 nm) demonstrated good results in *in vivo* photoacoustic (PA) imaging, MRI and enhanced cellular uptake (Jin et al., 2018; Figure 3).

### Biomedical Applications

**Scheme 1:**
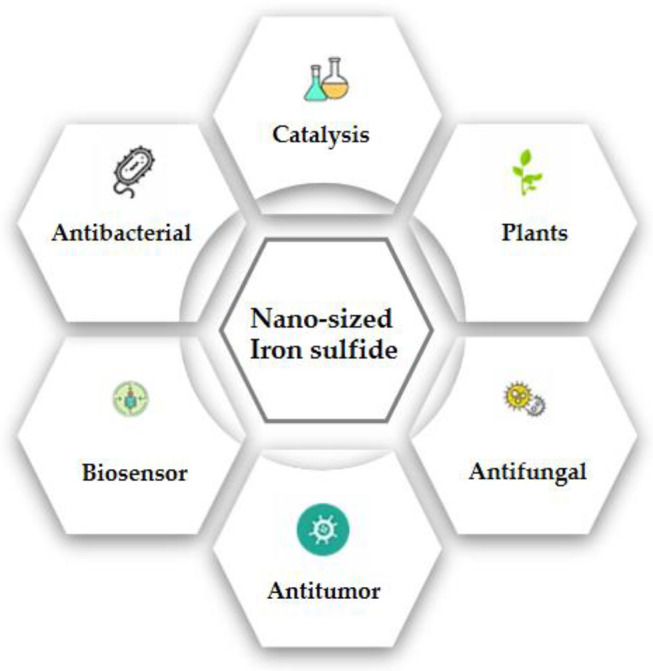
Schematic diagram of biomedical applications of nano-sized iron sulfide.

To-date, a variety of biomedical applications of iron sulfide (catalysts, antibacterial agents, cancer therapies, drug delivery systems, thrombolytic agents, biosensors, antifungal agents, seed improvers in phyto-applications) and their functional mechanisms have been summarized (Scheme 1).

### Enzyme-Like Catalysis

**Figure 4:**
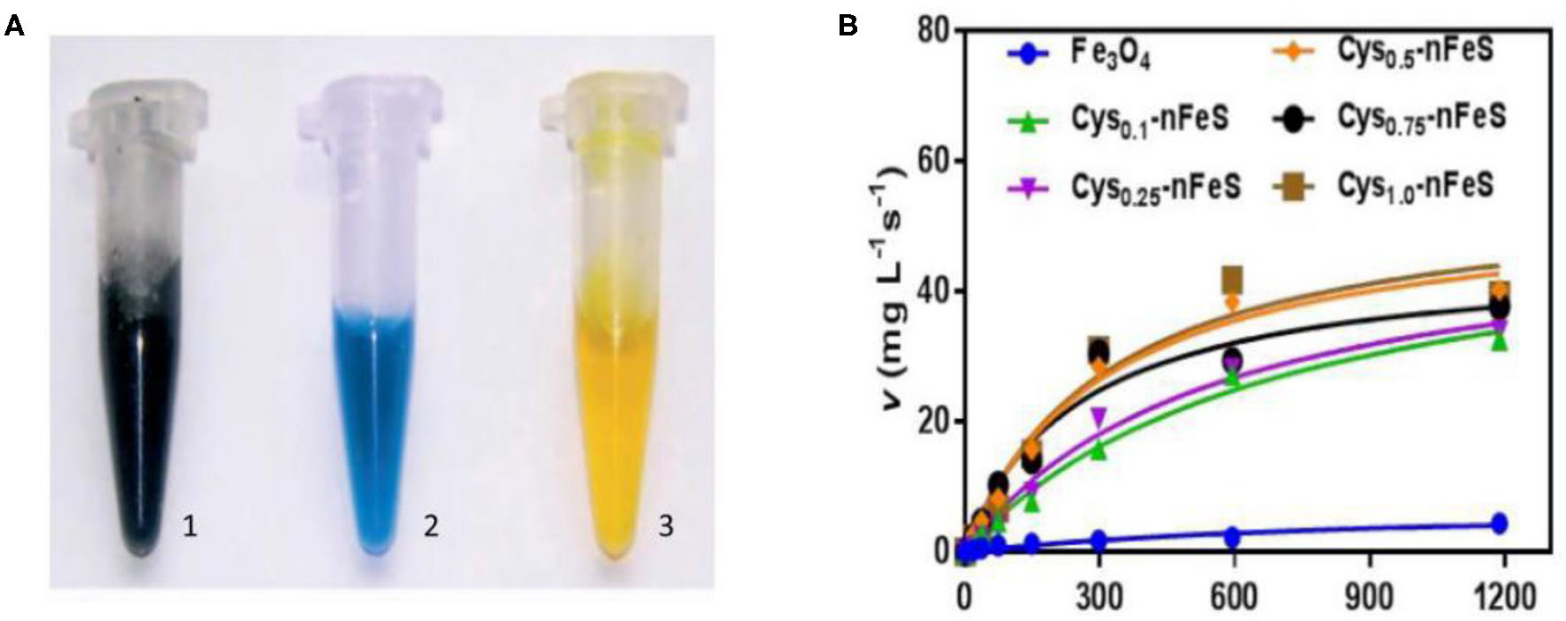
Schematic diagram of catalysis of iron sulfide as a nanozyme. **(A)** The peroxidase-like activity of FeS. Images of the suspension of sheet-like FeS nanostructure (1), mixture of TMB and H_2_O_2_ after catalytic reaction by sheet-like FeS nanostructure (2), mixture of TMB and H_2_O_2_ after adding H_2_SO_4_ to quench the catalytic reaction by sheet-like FeS nanostructure (3). Reproduced with permission from Dai et al. (2009). Copyright 2009, Wiley-VCH Verlag GmbH & Co. KGaA, Weinheim. **(B)** The catalase-like activity of Cys-nFeS. The trend of K*M* and the ratio of V*max*/K*M* in the kinetics assay with varied cysteine. Reproduced with permission from Xu et al. (2018). Copyright 2018, Nature Publishing Group.

Iron sulfur clusters are critical cofactors in many enzymes and proteins which conduct redox reactions and regulate oxidative stress. Thus, nano-sized iron sulfides are expected to perform similar catalysis and act as nanozymes. Previous studies have shown that iron sulfide can effectively activate persulfate (PS) or peroxymonosulfate (PMS) to generate sulfate radicals (Xiao et al., 2020). Other common reactions involving iron sulfide are shown in section Sonochemical Synthesis. Since iron oxide nanoparticles were shown to possess intrinsic peroxidase activity in 2007 (Gao et al., 2007), it has been speculated that iron sulfide has similar properties. The catalytic processes of iron sulfide are shown in Figure 4. High catalytic activity, multi-enzymes activities, harsh environmental resistance, storage stability, and the intrinsic advantages of nanomaterials provide further possibilities for biomedicine development, meaning that good alternatives to natural enzymes exist. The enzymatic activity of iron sulfide has been intensely investigated. In 2010, FeS nanosheets were reported to possess peroxidase-like activity. FeS suspensions were shown to catalyze the oxidation of peroxidase substrates, 3, 3′, 5, 5′-Tetramethylbenzidine (TMB) to produce a blue product in the presence of H_2_O_2_ (Dai et al., 2009) (Figure 4). Fe_7_S_8_ nanowires' also possessing intrinsic peroxidase activity was also reported in 2013 for which catalytic behavior was observed. The apparent K_*M*_ of Fe_7_S_8_ with TMB as a substrate was 0.548 mM, almost six times lower than that of horseradish peroxidase (HRP). These results demonstrate that Fe_7_S_8_ has a higher affinity to TMB than to HRP (Yao et al., 2013). In 2015, magnetic Fe_3_S_4_ NPs was shown to possess peroxidase-like activity. Through investigating steady state kinetics, Fe_3_S_4_ was shown to possess a higher affinity for H_2_O_2_ than HRP. The reasons could be that Fe_3_S_4_ binds to and reacts with H_2_O_2_, following which nanozyme is released prior to reacting with the second substrate TMB (Ding et al., 2016). nFeS (Fe_1−x_S and Fe_3_S_4_) (detailed description in section Hydrothermal Synthesis) have been shown to possess both peroxidase-like and catalase-like activity (Figure 4). nFeS is able to decompose H_2_O_2_ into free radicals and O_2_, promoting the release of polysulfanes (Xu et al., 2018). FeS_2_ has also been shown to possess amylase-like properties (Srivastava et al., 2014b).

### Antibacterial Alternatives

**Figure 5:**
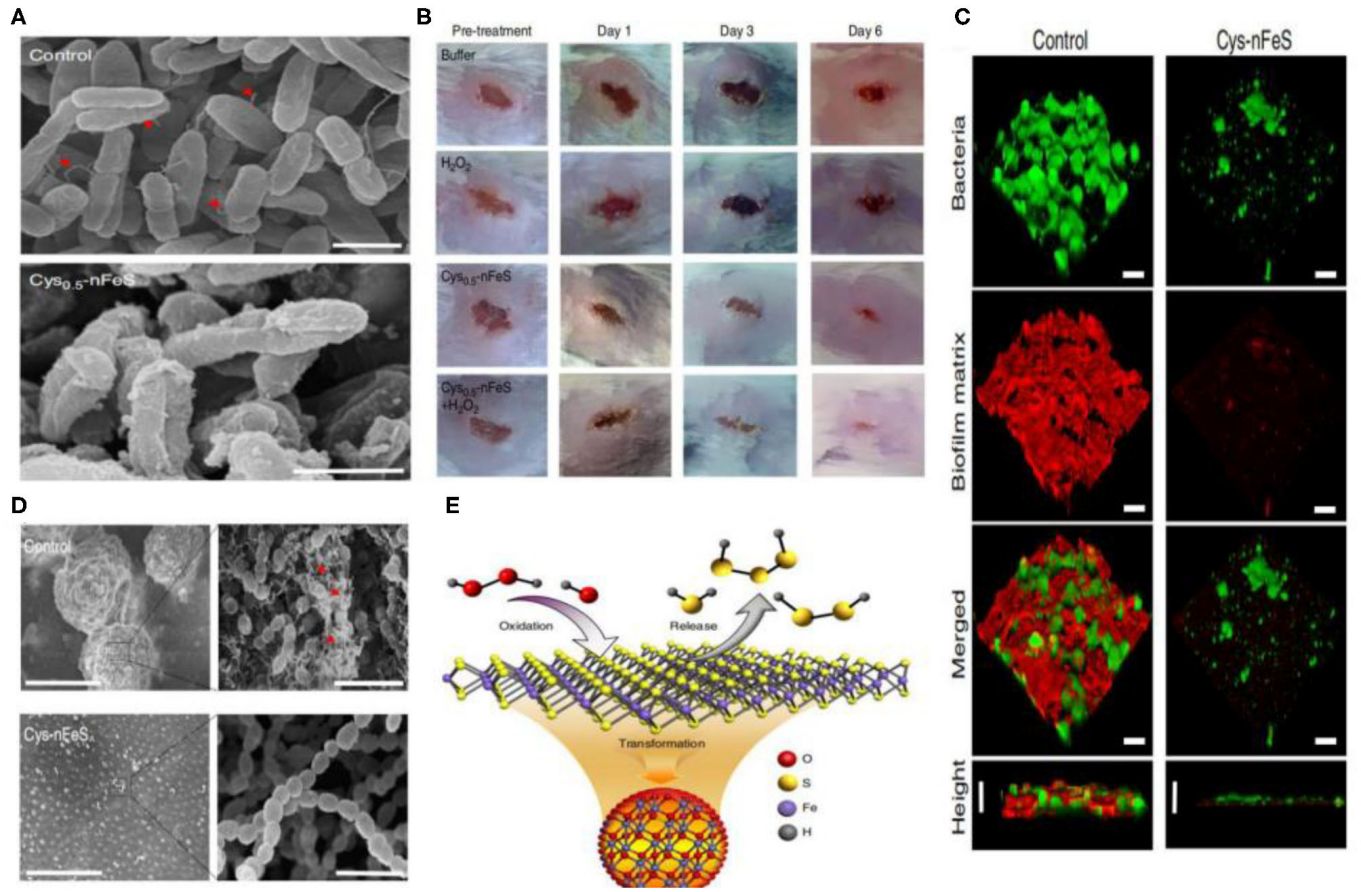
**(A)** Morphology of *P. aeruginosa* before (control) and after Cys-nFeS treatment. The red triangles indicate flagella. Scale bars: 1 μm. **(B)** Photographs of *P. aeruginosa* infected wounds treated with buffer (control), Cys-nFeS, H_2_O_2_, and Cys-nFeS + H_2_O_2_ at different times (five mice in each group). **(C)** Confocal 3D image of a *S. mutans* UA159 biofilm treated by Cys-nFeS. Scale bars: 100 μm. **(D)** SEM image of a *S. mutans* biofilm treated by Cys-nFeS. The red arrows indicate EPS. Left scale bars: 100 μm. Right scale bars: 3 μm. **(E)** Scheme of polysulfane release from nFeS. Reproduced with permission from Xu et al. (2018). Copyright 2018, Nature Publishing Group.

Schoonen et al. reported on the antibacterial action of FeS_2_. Its mechanism was shown to be related to the formation of H_2_O_2_. The same group also reported how minerals can induce the formation of reactive oxygen species (Cohn et al., 2006; Schoonen et al., 2006). The rapid absorption of Fe^2+^ can influence bacterial metabolism. The oxidization of Fe^2+^ to Fe^3+^ leads to reactive oxygen species (ROS) production and biomolecular damage. As a result, iron sulfide can act as antimicrobial agents. Iron sulfides have been reported to be effective in bacterial infections. In 2013, He et al. reported on the bacteriostatic activity of Fe_3_S_4_/Ag against *E. coli* (86.2%) and *S. aureus* (90.6%). Meanwhile, Fe_3_S_4_ NPs without Ag have no effect (He et al., 2013). The Arenas-Arrocena group synthesized Fe_x−1_S NPs and reported their antibacterial and cytotoxic activity in 2018. Antibacterial activity against *S. aureus, E. coli* and *E. faecalis* was observed (Argueta-Figueroa et al., 2018). In addition, Gao and coworkers discovered antibacterial inorganic iron polysulfides materials that were converted by organosulfur compounds in 2018. Inorganic nano-sulfides (nFeS, Fe_1−x_S, and Fe_3_S_4_) have shown inhibition against *Pseudomonas aeruginosa* and *Staphylococcus aureus*. These studies also described new strategies to synthesize iron sulfide nanomaterials. Furthermore, the *S. mutans* biofilm-related infections could be prevented by nFeS (Xu et al., 2018) (Figure 5). The antibacterial properties of FeS_2_-Bi_2_O_3_ against the pathogenic microorganisms *Mycobacterium tuberculosis* and *Salmonella* have also been measured (Manafi et al., 2019). Diksha et al. reported that FeS NPs could enhance intrabacterial ROS levels in bacteria by light irradiation in 2020. This was revealed as the primary antibacterial mechanism of FeS NPs (Agnihotri et al., 2020).

### Cancer Therapy

**Figure 6:**
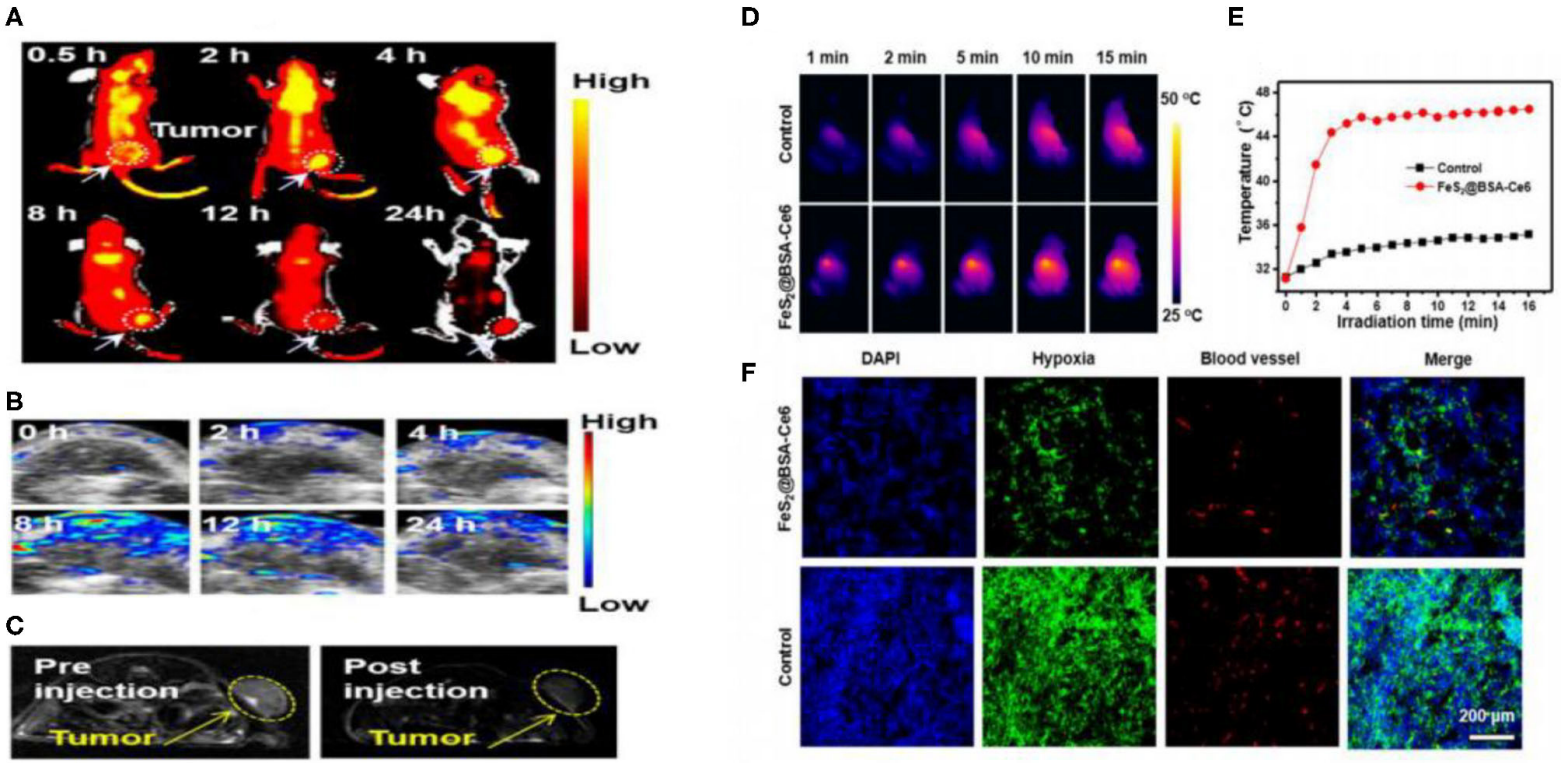
**(A)** *In vivo* fluorescence images of 4T1 tumor-bearing nude mice taken at different time points post iv injection of FeS_2_@BSA-Ce6 nanodots (3.5 mg/kg Ce6 and 12 mg/kg FeS_2_). **(B)** Photoacoustic images of tumors in mice taken at different time points post iv injection of FeS_2_@BSA-Ce6 nanodots. **(C)** MR images of 4T1 tumor-bearing nude mice before and 8 h after iv injection of FeS_2_@BSA-Ce6 nanodots. (3.5 mg/kg Ce6 and 12 mg/kg FeS_2_). **(D)** IR thermal images of tumors in mice iv injected with FeS_2_@BSA-Ce6 under 808 nm laser irradiation (0.8W/cm^2^, 15 min). **(E)** Surface temperature changes of tumors monitored by the IR thermal camera during laser irradiation. **(F)** Representative immunofluorescence images of tumor slices after hypoxia staining. The cell nucleus, hypoxia areas, and blood vessels were stained with DAPI (blue), antipimonidazole antibody (green), and anti-CD31 antibody (red), respectively. Reproduced with permission from Jin et al. (2018). Copyright 2017, American Chemical Society.

Chen and colleagues reported that tumors in mice could disturb iron metabolism in the major organs. The chemical form of iron in the tumors was ferrous-sulfide-like iron and ferritin, highlighting the potential of iron sulfide for cancer treatment (Chen and Chen, 2017). Chang et al. synthesized Fe_3_S_4_ particles with magnetic properties through co-precipitation. The NPs were used for cancer hyperthermia providing a new avenue for multimodal anticancer therapies (Chang et al., 2011). In 2015, Yang et al. concluded that triangle nanoplates (FeS/PEG nanostructures) could be used as nanoagents for *in vivo* MRI-guided photothermal cancer treatment. High doses of FeS nanoplates were shown to be safe and effective in mice. This study highlighted the potential clinical use of FeS, for MRI in addition to PTT (Figure 6) (Yang et al., 2015). In 2018, Fe_3_S_4_ nanosheets were shown to possess high efficiencies for MRI guided photothermal and chemodynamic synergistic therapy, opening up a new direction for the design of inorganic iron sulfide for future clinical applications (Guan et al., 2018). Tiny nano-sized iron sulfide with simple biomineralization method has also been attention owing to its huge potential *in vivo* application especially in cancer therapy combined with its excellent photothermal and magnetic performance. FeS_2_@BSA-Ce6 (detailed in section Biomineralization) exhibited good results whether *in vivo* multimodal imaging or *in vivo* combined therapy (Jin et al., 2018). Meanwhile, the latest literature proved 3 nm FeS@BSA QDs could be used as T1-weighted MRI contrast agents. Moreover, the ultrasmall QDs showed good results in photothermal therapy and they could be cleared via glomerular filtration into bladder after treatment (Yang et al., 2020). The use of iron chalcogenides has also been investigated. Cu_5_FeS_4_-PEG NPs were effective in dual-modal imaging and PTT (Zhao et al., 2016). In 2017, CuFeS_2_ nanoplates were used for *in vivo* photothermal/photoacoustic imaging and cancer chemotherapy/PTT (Ding et al., 2017).

### Drug Delivery

**Figure 7:**
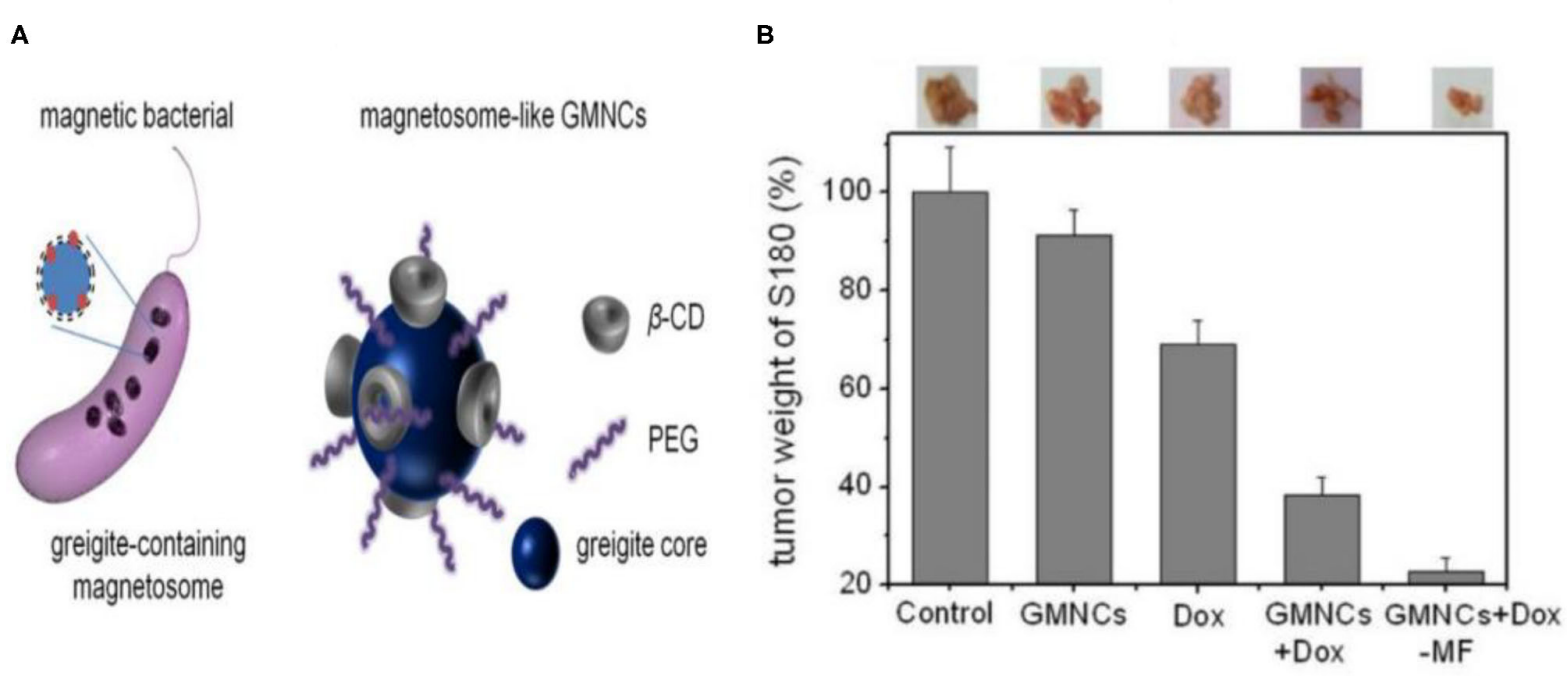
**(A)** Schematical illustration of the greigite-containing magnetosome and the chemical synthesized magnetosome-like GMNCs. **(B)** Growth inhibition effect in murine S180 sarcoma model of the sample. The photos and weight ratios of tumor tissue from mice treated with normal saline (control group), GMNCs, Dox at low concentration, and GMNCs loading with Dox (without and with the guidance of external magnetic field), respectively. Reproduced with permission from Feng et al. (2013). Copyright 2013, Nature Publishing Group.

Iron sulfide holds utility as a drug carrier. In previous studies, modified β-cyclodextrin (β-CD) and PEG Fe_3_S_4_ (GMNCs) were used as drug loading NPs. Both β-CD and PEG have been used to control the shape and size of GMNCs as surfactants. In addition, the biocompatibility of Fe_3_S_4_ is enhanced, with entrapment efficiencies of 58.7% for the modified delivery of the chemotherapeutic drug doxorubicin. Meanwhile, the enhanced chemotherapeutic treatment of mouse tumors was obtained through the intravenous injection of doxorubicin (Dox) loaded GMNCs (Feng et al., 2013; Figure 7).

### Thrombolytic Agents

**Figure 8:**
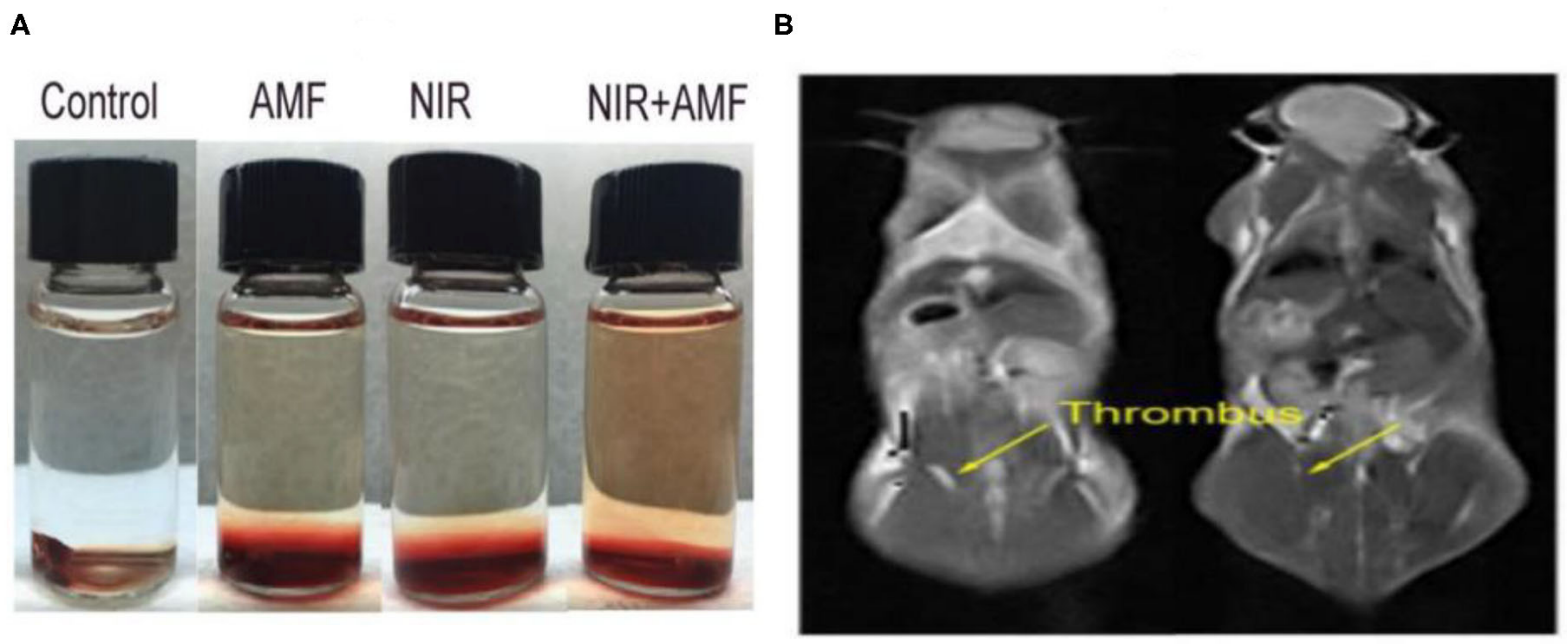
**(A)** Thrombolytic capacity *in vitro* of Fe_3_S_4_ nanoparticles under the indicated conditions. Fe_3_S_4_ nanoparticles: 0.5 mg/mL; NIR: 808 nm, 0.33 W cm^−2^; AMF: 4.2 × 10^9^ A m^−1^ s^−1^. **(B)** T2-weighted MR imaging *in vivo* of a mouse with celiac vein thrombosis before (left) and after (right) an intravenous injection of a solution of the Fe_3_S_4_ nanoparticles followed by the simulation of AMF. Reproduced with permission from Fu et al. (2019). Copyright 2019, Frontiers in Materials.

In studies of vascular disease, the removal of thrombosis through non-invasive methods is challenging. However, studies regarding iron sulfide NPs as thrombolytic agents have been reported. Ge et al. first highlighted the utility of Fe_3_S_4_ NPs as thrombolytic agents with both photothermal and magnetothermal thrombolytic capability. Using Fe_3_S_4_ nanoparticles, celiac vein thrombosis could be prevented using magnetic hyperthermia combined PTT. Both *in vivo* and *in vitro*, Fe_3_S_4_ has demonstrated beneficial effects for the removal of thrombi (Figure 8), providing a novel hyperthermia strategy for the prevention of thrombosis (Fu et al., 2019).

### Biosensors

Iron sulfides have been used as glucose sensors due to their intrinsic peroxidase-like activity (Dai et al., 2009). Glucose sensors can be developed using colorimetric methods in which cascade reactions form the core mechanism of glucose detection. TMB could be oxidized to oxTMB in the presence of glucose, GOx and iron sulfide. H_2_O_2_ produced by the decomposition of glucose in the presence of glucose oxidase can be used as a substrate for iron sulfide. Iron sulfide peroxidase-like mimics can oxidize TMB to oxTMB in the presence of H_2_O_2_. Fe_7_S_8_ nanowires also possess peroxidase activity. Using a linear range, glucose concentrations of 5 × 10^−6^ to 5 × 10^−4^ M could be detected (Yao et al., 2013). In 2016, Xian and colleagues used Fe_3_S_4_ magnetic nanoparticles (MNPs) to quantify glucose concentrations in human serum. A linear range was measured from 2 to 100 μM, and the limit of detection (LOD) was 0.16 μM (Ding et al., 2016). These studies highlighted the potential of as-prepared iron sulfide as both glucose sensors and artificial peroxidase nanozymes.

### Antifungal Agents

*In vitro* antifungal FeS NPs exhibited significant anti-fungal activity against *F. verticillioides* at 18 μg ml^−1^, with a higher efficiency than standard fungicides (carbendazim (median effective dose (ED50): 230 μg ml^−1^). These were the first reports highlighting the antifungal activity of iron sulfide. The influence of FeS on both seed health and quality parameters of rice was also evaluated based on this antifungal activity. Iron sulfides were shown to be effective in iron deficient soils as an alternative to high dose organic fungicides (Ahuja et al., 2019).

### Seed Improvements in Phyto-Applications

**Figure 9:**
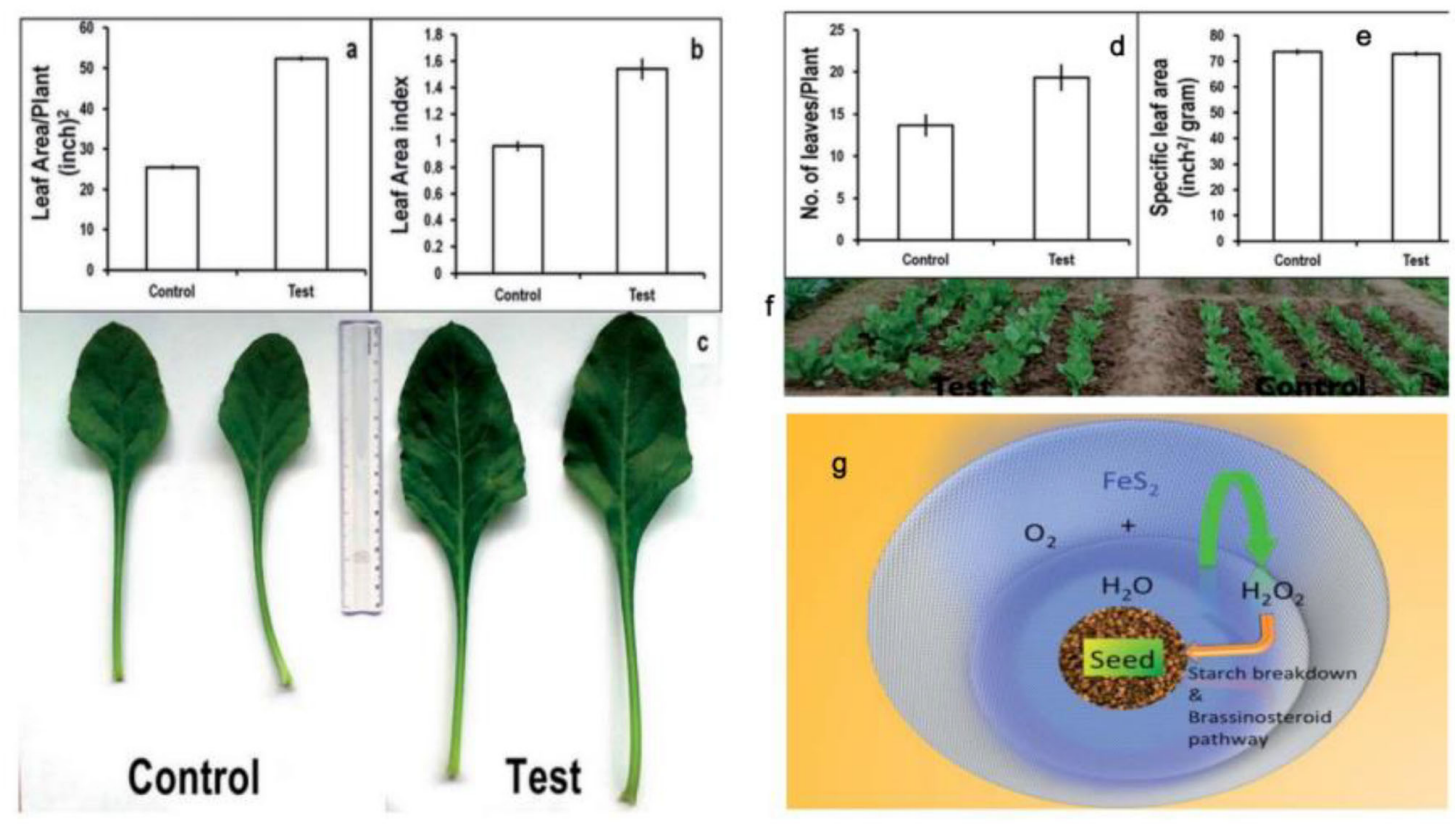
Plant growth parameters: control vs. test (FeS_2_). **(a)** Leaf area/Plant, showing significant increase in leaf area/test plants (52.4 ± 0.3) as compared to control (25.6 ± 0.2). **(b)** Leaf area index signifying total photosynthetic area available to plant and high values for test samples (1.5 ± 0.07) can be correlated with high biomass content of pro-fertilized spinach plants in comparison with (0.9 ± 0.02). **(c)** Comparative photograph of leaves showing larger leaf area in test plants as compared to control plants. Plant growth parameters: control vs. test (FeS_2_). **(d)** Number of leaves/Plant: control: 13 ± 1.0; test: 19 ± 1.0. **(e)** Specific leaf area signifies leaf thickness and was found similar for both test and control samples. **(f)** Field photograph taken at day 50 (just before harvesting the crop) depicting that the test group plants have comparatively more foliage as compared to the control plants. **(g)** Proposed outline of the mechanism of action of FeS_2_ on spinach seed in enhancing germination and plant growth. Reproduced with permission from Srivastava al. (2014). Copyright 2014, The Royal Society of Chemistry.

FeS_2_ represents a photovoltaic material, which increases plant biomass in the seeds of chickpeas (Cicer arietinum). Meanwhile, the mechanism of functional FeS_2_ is attributed to its amylase-like activity. In the presence of H_2_O and FeS_2_, starch in the seeds can be broken down to H_2_O_2_, which participates in the absorption of CO_2_ and improves plant health. Spinach seeds treated with FeS_2_ exhibited broader leaf morphologies, larger leaf numbers and an increased biomass (Srivastava et al., 2014b) (Figure 9). In later studies, seed priming with FeS_2_ was reported as an innovative strategy (Das et al., 2016). FeS_2_ also improved both seed yield and growth in the *Brassica juncea* field (Rawat et al., 2017).

## Conclusions

In summary, we have highlighted the most recent methods of nano-iron sulfide synthesis, including nano iron sulfide modifications and characterizations. Strikingly, nano-sized iron sulfides demonstrate versatile physiochemical properties, enzyme-like catalysis, high stability and biocompatibility, which facilitate their biomedical applications. A range of nano-iron sulfides have been assessed in catalysis, tumor therapy, antibacterials and antifungals, drug delivery, biosensors, thrombus removal and in plants. Their advantages include (1) high biocompatibility due to the key role of iron and sulfur in natural life; (2) the photothermal and magnetic properties of nano-sized iron sulfide; (3) their nanostructure and large surface area that can improve drug delivery; and (4) their enzyme-like properties as nanozymes, including their high reactivity to numerous chemical substances that can regulate hydrogen peroxide, ROS and various catalytic reactions to treat related diseases.

**Scheme 2:**
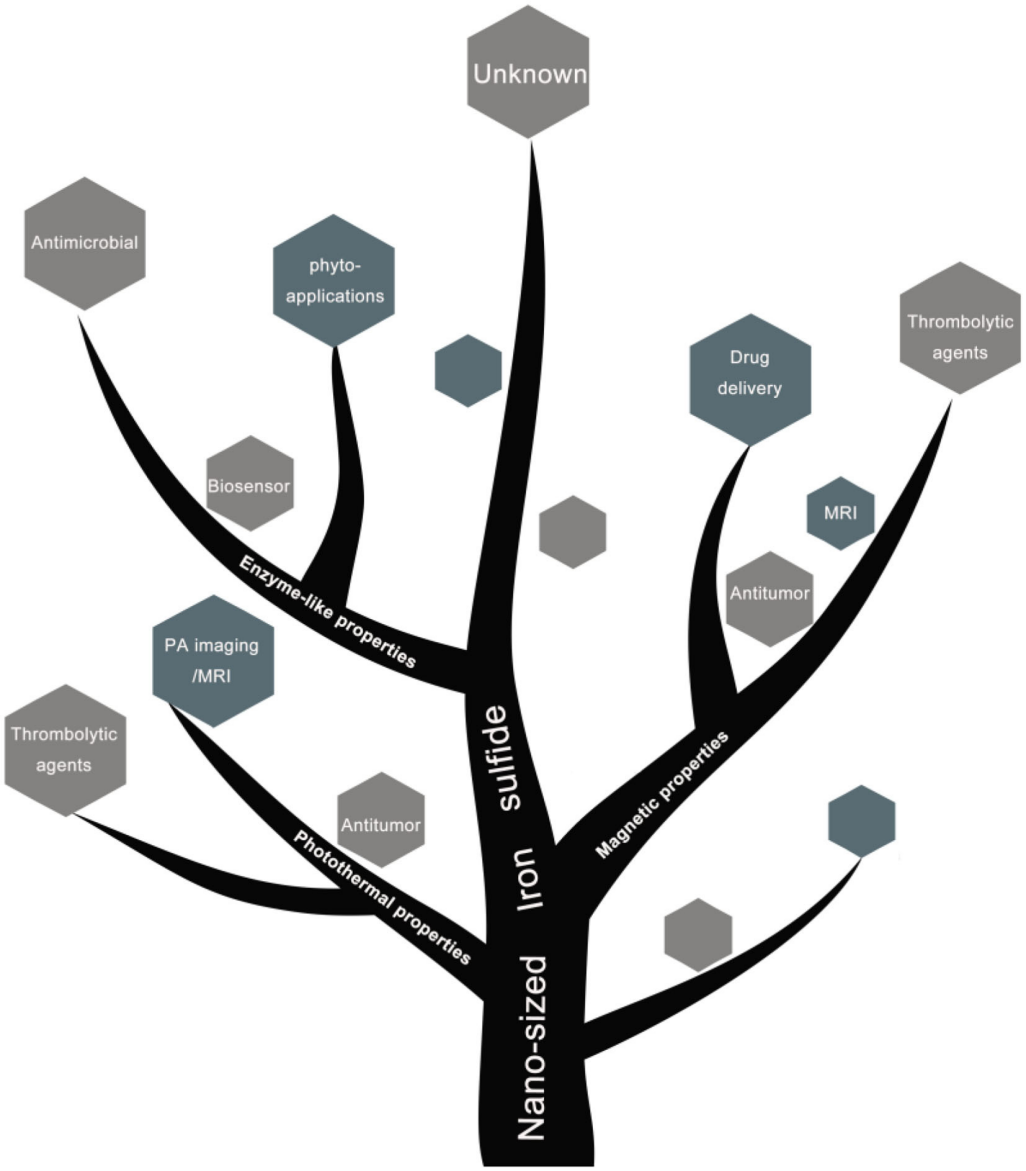
Schematic diagram of biomedical applications based on physiochemical properties of nano-sized iron sulfide.

Although it has been shown that nano-sized iron sulfides represent great potential in numerous applications in biomedicine (Scheme 2), a number of issues remain to be addressed, including the synthesis of iron sulfide that is stable and in a single phase, with modifications to adapt to the biological environment. Studies have found that the modification of molecular CTAB inhibits the preparation of Fe_3_S_4_ NPs due to competitive inhibition with Na_2_S under acidic conditions, resulting in the formation of non-magnetic iron sulfides and other byproducts. Citrate modified nanoparticles have not sufficient particle spacing due to aggregation effects (Simeonidis et al., 2016). In addition, the biomedical assessments of iron sulfides remain sparse, and their mechanisms of action under physiological conditions are poorly understood. The intrinsic enzyme-like properties of iron sulfide as nanozymes may provide a window to understand its biological effects and potential cytotoxicity *in vivo*. Taken together, nano-sized iron sulfides possess versatile physiochemical properties and enzyme-like properties, which describe a form of distinctive nanomaterials with great potential for use in biomedical applications.

## Author Contributions

All authors listed have made a substantial, direct and intellectual contribution to the work, and approved it for publication.

## Conflict of Interest

The authors declare that the research was conducted in the absence of any commercial or financial relationships that could be construed as a potential conflict of interest.

## References

[B1] Agnihotri S. Mohan T. Jha D. Gautam H. Roy I. (2020). Dual Modality FeS Nanoparticles with Reactive Oxygen Species-Induced and Photothermal Toxicity toward Pathogenic Bacteria. ACS OMEGA 5, 597–602. 10.1021/acsomega.9b03177 31956807 PMC6964285

[B2] Ahuja R. Sidhu A. Bala A. (2019). Synthesis and evaluation of iron(ii) sulfide aqua nanoparticles (FeS-NPs) against Fusarium verticillioides causing sheath rot and seed discoloration of rice. Eur. J. Plant Pathol. 155, 163–171. 10.1007/s10658-019-01758-3

[B3] Argueta-Figueroa L. Martinez-Alvarez O. Santos-Cruz J. Garcia-Contreras R. Acosta-Torres L. S. Fuente-Hernández J.. (2017). Nanomaterials made of non-toxic metallic sulfides: a systematic review of their potential biomedical applications. Mater. Sci. Eng. C 76, 1305–1315. 10.1016/j.msec.2017.02.120 28482499

[B4] Argueta-Figueroa L. Torres-Gómez N. García-Contreras R. Vilchis-Nestor A. R. Martínez-Alvarez O. Acosta-Torres L. S. (2018). Hydrothermal synthesis of pyrrhotite (Fe_x−1_S) nanoplates and their antibacterial, cytotoxic activity study. Progress Nat. Sci. 28, 447–455. 10.1016/j.pnsc.2018.06.003

[B5] Barand A. Russo S. (2009). Modeling the environmental stability of FeS_2_ nanorods, using lessons from biomineralization. Nanotechnology 20:115702. 10.1088/0957-4484/20/11/115702 19420450

[B6] Barnard A. Russo S. (2009). Morphological stability of Pyrite FeS_2_ nanocrystals in water. J. Phys. Chem. C 113, 5376-5380. 10.1021/jp809377s

[B7] Beal J. Etchegoin P. Tilley R. (2012). Synthesis and characterisation of magnetic iron sulfide nanocrystals. J. Solid State Chem. 189, 57–62. 10.1016/j.jssc.2012.01.015

[B8] Bi Y. Yuan Y. Exstrom C. L. Darveau S. A. Huang J. (2011). Air stable, photosensitive, phase pure iron pyrite nanocrystal thin films for photovoltaic application. Nano Letter 11, 4953–4957. 10.1021/nl202902z 21992489

[B9] Chang Y. Savitha S. Sadhasivam S. Hsu C. Lin F. (2011). Fabrication, characterization, and application of greigite nanoparticles for cancer hyperthermia. J. Colloid Interface Sci. 363, 314–319. 10.1016/j.jcis.2010.06.069 21824623

[B10] Chen R. Chen G. (2017). Tumor-induced disorder of iron metabolism in major organs: a new insight from chemical speciation of iron. J. Int. Med. Res 46, 70–78. 10.1177/0300060517718711 28718696 PMC6011321

[B11] Chen B. Gu N. (2017). Current status and development of pharmaceutical iron based nanomaterials. Mater. China 36, 211–218.

[B12] Cohn C. Laffers R. Simon S. O'Riordan T. Schoonen M. (2006). Role of pyrite in formation of hydroxyl radicals in coal: possible implications for human health. Part. Fibre Toxicol. 3:16. 10.1186/1743-8977-3-16 17177987 PMC1764420

[B13] Dai Z. Liu S. Bao J. Ju H. (2009). Nanostructured FeS as a mimic peroxidase for biocatalysis and biosensing. Chem. Eur. J. 15, 4321–4326. 10.1002/chem.200802158 19267381

[B14] Das C. Srivastava G. Dubey A. Roy M. Jain S. Sethy N. (2016). Nano-ironpyrite seed dressing: A sustainable intervention to reduce fertilizer consumption in vegetable (beetroot, carrot), spice (fenugreek), fodder (alfalfa), and oilseed (mustard, sesamum) crops. Nanotechnol. Environ. Eng. 1, 1–12. 10.1007/s41204-016-0002-7

[B15] Davison W. (1991). The solubility of iron sulphides in synthetic and natural waters at ambient temperature. Aquatic Sci. 53, 309–329. 10.1007/BF00877139

[B16] De Leeuw N. Parker S. Sithole H. Ngoepe P. E. (2000). Modeling the surface structure and reactivity of pyrite: introducing a potential model for FeS_2_. J. Phys. Chem. B 104, 7969–7976. 10.1021/jp0009498

[B17] Ding B. Yu C. Li C. Deng X. Ding J. Cheng Z.. (2017). Cis-platinum pro-drug-attached CuFeS_2_ nanoplates for *in vivo* photothermal/photoacoustic imaging and chemotherapy/photothermal therapy of cancer. Nanoscale 9, 16937–16949. 10.1039/C7NR04166G 29077118

[B18] Ding C. Yan Y. Xiang D. Zhang C. Xian Y. (2016). Magnetic Fe_3_S_4_ nanoparticles with peroxidase-like activity, and their use in a photometric enzymatic glucose assay. Microchim. Acta 183, 625–631. 10.1007/s00604-015-1690-6

[B19] Dong H. Fan Y. Zhang W. Gu N. Zhang Y. (2019). Catalytic mechanisms of nanozymes and their applications in biomedicine. Bioconjug. Chem. 30, 1273–1296. 10.1021/acs.bioconjchem.9b00171 30966739

[B20] Elliot A. (2010). Structure of pyrrhotite 5c (Fe_9_S_10_). Acta Crystallogr. Section B 66, 271–279. 10.1107/S0108768110011845 20484798

[B21] Esmaeili E. Eslami-Arshaghi T. Hosseinzadeh S. Elahirad E. Jamalpoor Z. Hatamie S.. (2020). The biomedical potential of cellulose acetate/polyurethane nanofibrous mats containing reduced graphene oxide/silver nanocomposites and curcumin: antimicrobial performance and cutaneous wound healing. Int. J. Biol. Macromol. 152, 418–427. 10.1016/j.ijbiomac.2020.02.295 32112830

[B22] Feng M. Lu Y. Yang Y. Zhang M. Xu Y. Gao H.. (2013). Bioinspired greigite magnetic nanocrystals: chemical synthesis and biomedicine applications. Sci. Rep. 3:994. 10.1038/srep02994 24141204 PMC3801133

[B23] Fleet M. (1971). The crystal structure of a pyrrhotite (Fe_7_S_8_). Acta Cryst. B 27:1864 10.1107/S0567740871004990

[B24] Fu D. Liu J. Ren Q. Ding J. Ding H. Chen X. (2019). magnetic iron sulfide nanoparticles as thrombolytic agents for magnetocaloric therapy and photothermal therapy of thrombosis. Front. Mater. 6:316 10.3389/fmats.2019.00316

[B25] Gan Y. Xu F. Luo J. Yuan H. Jin C. Zhang L. (2016). One-pot biotemplate synthesis of FeS_2_ decorated sulfur-doped carbon fiber as high capacity anode for lithium-ion batteries. Electrochim. Acta 209, 201–209. 10.1016/j.electacta.2016.05.076

[B26] Ganbaatar N. Matsuzaki N. Nakazawa Y. Afrin R. Aono M. Yano T. (2016). Surface force analysis of pyrite (FeS_2_): its reactivity to amino acid adsorption. Adv. Mater. Phys. Chem. 6, 167–176. 10.4236/ampc.2016.67018

[B27] Gao L. Zhuang J. Nie L. Zhang J. Zhang Y. Gu N.. (2007). Intrinsic peroxidase-like activity of ferromagnetic nanoparticles. Intrinsic peroxidase-like activity of ferromagnetic nanoparticles. Nat. Nanotechnol. 2, 577–583. 10.1038/nnano.2007.260 18654371

[B28] Gao S. Huang F. Song D. Li G. Liu Q. Feng T. (2015). Growth mechanism and stability study on the Fe_3_S_4_ nanocrystals synthesized under thermal and humid conditions, in Proceedings of the 11th International Congress for Applied Mineralogy. 10.1007/978-3-319-13948-7_13

[B29] Guan G. Wang X. Li B. Zhang W. Cui Z. Lu X.. (2018). Transformed” Fe_3_S_4_ tetragonal nanosheets: a high-efficiency and body-clearable agent for magnetic resonance imaging guided photothermal and chemodynamic synergistic therapy. Nanoscale 10, 17902–17911. 10.1039/C8NR06507A 30226246

[B30] Guo S. Li J. Ma Z. Chi Y. Xue H. (2016). A facile method to prepare FeS/porous carbon composite as advanced anode material for lithium-ion batteries. J. Mater. Sci. 52, 2345–2355. 10.1007/s10853-016-0527-y29655282

[B31] Guo Z. Sun F. Han B. Lin K. Zhou L. Yuan W. (2017). Iron vacancy in tetragonal Fe_1−x_S crystals and its effect on the structure and superconductivity. Phys. Chem. Chem. Phys. 19, 9000–9006. 10.1039/C7CP00068E 28303269

[B32] He Q. Huang C. Liu J. (2013). Preparation, Characterization and antibacterial activity of magnetic greigite and Fe_3_S_4_/Ag nanoparticles. Nanosci. Nanotechnol. Lett. 5, 1–8. 10.1166/nnl.2014.1727

[B33] He Y. Wilson J. Su C. Wilkin R. T. (2015). Review of abiotic degradation of chlorinated solvents by reactive iron minerals in aquifers. Ground Water Monitoring Remediation 35, 57–75. 10.1111/gwmr.12111

[B34] Jin A. Mi-Ju K. Kug-Seung L. Yu S. Sung Y. (2019). Spindle-like Fe_7_S_8_/N-doped carbon nanohybrids for high-performance sodium ion battery anodes. Nano Res. 12, 695–700. 10.1007/s12274-019-2278-y

[B35] Jin J. Wu W. Min H. Wu H. Wang S. Ding Y. (2017). A glassy carbon electrode modified with FeS nanosheets as a highly sensitive amperometric sensor for hydrogen peroxide. Microchim. Acta 184, 1389–1396. 10.1007/s00604-017-2105-7

[B36] Jin Q. Liu J. Zhu W. Dong Z. Liu Z. Cheng L. (2018). Albumin-assisted synthesis of ultrasmall FeS_2_ nanodots for imaging-guided photothermal enhanced photodynamic therapy. ACS Appl. Mater. Interfaces 10, 332–340. 10.1021/acsami.7b16890 29220162

[B37] Kar S. Chaudhuri S. (2004). Solvothermal synthesis of nanocrystalline FeS_2_ with different morphologies. Chem. Phys. Lett. 398, 22–26. 10.1016/j.cplett.2004.09.028

[B38] Kasting J. (1993). Earth's early atmosphere. Science 259, 920–926. 10.1126/science.11536547 11536547

[B39] Kavner A. Duffy T. Shen G. (2001). Phase stability and density of FeS at high pressures and temperatures: implications for the interior structure of Mars. Earth Planet. Sci. Lett. 185, 25–33. 10.1016/S0012-821X(00)00356-317797532

[B40] Kim E. J. Batchelor B. (2009). Synthesis and characterization of pyrite (FeS2) using microwave irradiation. Mater. Res. Bull. 44, 1553–1558. 10.1016/j.materresbull.2009.02.006

[B41] Kong X. Lou T. Li Y. (2005). Fe_7_S_8_ nanorods and nanosheets. J. Alloys Compd. 390, 236–239. 10.1016/j.jallcom.2004.07.054

[B42] Kuhn S. J. Eskildsen M. R. Debeer-Schmitt L. Li L. de La Cruz C. Sefat A. S. (2016). Structure and magnetic interactions in FeS: a low-Tc superconductor. Bull. Am. Phys. Soc. 61, 2. Abstracts retrieved from APS March Meeting (Abstract ID: BAPS.2016.MAR.H11.12).

[B43] Kwon K. Refson K. Bone S. Qiao R. Yang W. Liu Z. (2011). Magnetic ordering in tetragonal FeS: evidence for strong itinerant spin fluctuations. Phys. Rev. B Cond. Matt. 83, 064402.1–064402.7. 10.1103/PhysRevB.83.064402

[B44] Lefèvre C. Abreu F. Lins U. Bazylinski D. (2010). Nonmagnetotactic multicellular prokaryotes from low-saline, nonmarine aquatic environments and their unusual negative phototactic behavior. Appl. Environ. Microbiol. 76, 3220–3227. 10.1128/AEM.00408-10 20363801 PMC2869151

[B45] Li M. Yao Q. Zhou G. Qu X. Mu C. Fu S. (2011). Microwave-assisted controlled synthesis of monodisperse pyrite microspherolites. Cryst. Eng. Comm. 13, 5936–5942. 10.1039/c1ce05478c

[B46] Li X. Xu H. Chen Z. Chen Z. Chen G. (2011). Biosynthesis of nanoparticles by microorganisms and their applications. J. Nanomater. 2011, 1–16. 10.1155/2011/27097421808638

[B47] Li Y. Pu Q. Li S. Zhang H. Wang X. Yao H. (2019). Machine learning methods for research highlight prediction in biomedical effects of nanomaterial application. Patt. Recognit. Lett. 117, 111–118. 10.1016/j.patrec.2018.11.008

[B48] Liang M. Yan X. (2019). Nanozymes: from new concepts, mechanisms, and standards to applications. Acc. Chem. Res. 52, 2190–2200. 10.1021/acs.accounts.9b00140 31276379

[B49] Ma J. Chang L. Lian J. Huang Z. Duan X. Liu X.. (2010). Ionic liquid-modulated synthesis of ferrimagnetic Fe_3_S_4_ hierarchical superstructures. Chem. Commun. 46, 5006–5008. 10.1039/c0cc00479k 20523925

[B50] Manafi A. Hosseini M. Fakhri A. Gupta V. Agarwal S. (2019). Investigation of photocatalytic process for iron disulfide-bismuth oxide nanocomposites by using response surface methodology: structural and antibacterial properties. J. Mol. Liq. 289:110950 10.1016/j.molliq.2019.110950

[B51] Mann S. Sparks N. Frankel R. Bazylinski D. Jannasch H. (1990). Biomineralization of ferrimagnetic greigite (Fe_3_S_4_) and iron pyrite (FeS_2_) in a magnetotactic bacterium. Nature 343, 258–261. 10.1038/343258a0

[B52] Mei B. Ma Z. (2013). Study of anti-friction performance of spherical FeS nanoparticle. Appl. Mechan. Mater. 475-476, 1334–1339. 10.4028/www.scientific.net/AMM.475-476.1334

[B53] Menyeh A. O'Reilly W. (1997). Magnetic hysteresis properties of fine particles of monoclinic pyrrhotite Fe_7_S_8_. J. Geomag. Geoelectr. 49, 965–976. 10.5636/jgg.49.965

[B54] Mofokeng T. Mabena G. Moloto M. J. Shumbula P. M. Mubiayi P. Nyamukamba P. (2017). Temperature influence on the lactose capped metal sulphide nanoparticles. Chalcogenide Lett. 14, 347–355.

[B55] Mohindar S. Jagadeesh M. (1979). Temperature-dependent magnetic susceptibility of marcasite (FeS_ {2}). Phys. Rev. B 20:3897 10.1103/PhysRevB.20.3897

[B56] Moore J. Nienhuis E. Ahmadzadeh M. Mccloy J. (2019). Synthesis of greigite (Fe_3_S_4_) particles via a hydrothermal method. AIP Adv. 9:035012 10.1063/1.5079759

[B57] Niño M. Flores E. Sanchez C. Rojo J. (2018). Reactivity of a FeS surface under room temperature exposure to nitrogen and H_2_S. J. Phys. Chem. B 122, 705–712. 10.1021/acs.jpcb.7b06309 28915037

[B58] Ono S. (2007). Magnetic phase transition of FeS at high pressures. Acta Crystallogr. Sect. A Found. Crystallogr. 63:59 10.1107/S0108767307098716

[B59] Paolella A. George C. Povia M. Zhang Y. Krahne R. Gich M. (2011). Charge transport and electrochemical properties of colloidal greigite (Fe_3_S_4_) nanoplatelets. Chem. Mater. 23, 3762–3768. 10.1021/cm201531h

[B60] Powell A. V. Vaqueiro P. Knight K. S. Chapon L. C. Sánchez R. D. (2004). Structure and magnetism in synthetic pyrrhotite Fe_7_S_8_: a powder neutron-diffraction study. Phys. Rev. B 70, 014415 10.1103/PhysRevB.70.014415

[B61] Qi W. Cowan J. (2011). Structural, mechanistic and coordination chemistry of relevance to the biosynthesis of iron–sulfur and related iron cofactors. Coord. Chem. Rev. 255, 688–699. 10.1016/j.ccr.2010.10.016 21499539 PMC3074115

[B62] Rawat M. Nayan R. Negi B. Zaidi M. G. H. Arora S. (2017). Physio-biochemical basis of iron-sulfide nanoparticle induced growth and seed yield enhancement in b. juncea. Plant Physiol. Biochem. 118, 274–284. 10.1016/j.plaphy.2017.06.021 28666234

[B63] Rickard D. (2006). The solubility of FeS. Geochim. Cosmochim. Acta 70, 5779–5789. 10.1016/j.gca.2006.02.029

[B64] Rickard D. Luther G. (2007). Chemistry of iron sulfides. Chem. Rev. 107, 514–562. 10.1021/cr0503658 17261073

[B65] Roberts A. (1995). Magnetic properties of sedimentary greigite (Fe_3_S_4_). Earth Planetary Sci. Lett. 134, 227–236. 10.1016/0012-821X(95)00131-U

[B66] SantosCarballal D. Roldan A. Dzade N. de Leeuw N. H. (2017). Reactivity of CO_2_ on the surfaces of magnetite (Fe_3_O_4_), greigite (Fe_3_S_4_) and mackinawite (FeS). Philos. Trans. R. Soc. A 376:65. 10.1098/rsta.2017.0065 29175834 PMC5719222

[B67] Scaini M. Bancroft G. Knipe S. (1998). Reactions of aqueous Au^1+^ sulfide species with pyrite as a function of pH and temperature. Am. Mineral. 83, 316–322. 10.2138/am-1998-3-415

[B68] Schoonen M. Cohn C. Roemer E. Laffers R. Simon S. O'Riordan T. (2006). Mineral-induced formation of reactive oxygen species. Med. Mineraol. Geochem. 64, 179–221. 10.2138/rmg.2006.64.7

[B69] Simeonidis K. Liébana-Viñas S. Wiedwald U. Ma Z. Li Z.-A. Spasova M. (2016). A versatile large-scale and green process for synthesizing magnetic nanoparticles with tunable magnetic hyperthermia features. RSC Adv. 6, 53107–53117. 10.1039/C6RA09362K

[B70] Snowball I. (1991). Magnetic hysteresis properties of greigite (Fe_3_S_4_) and a new occurrence in holocene sediments from swedish lappland. Phys. Earth Planetary Interiors 68, 32–40. 10.1016/0031-9201(91)90004-2

[B71] Srivastava G. Das A. Kusurkar T. Roy M. Airan S. Sharma R. (2014a). Ironpyrite, a potential photovoltaic material, increases plant biomass upon seed pretreatment. Mater. Express 4, 23–31. 10.1166/mex.2014.1139

[B72] Srivastava G. Das C. K. Das A. Singh S. Roy M. Kim H. (2014b). Seed treatment with iron pyrite (FeS_2_) nanoparticles increases the production of spinach. RSC Adv. 4, 58495–58504. 10.1039/C4RA06861K

[B73] Usher C. Paul K. Narayansamy J. Kubicki J. Sparks D. Schoonen M.. (2005). Mechanistic aspects of pyrite oxidation in an oxidizing gaseous environment: an *in situ* HATR–IR isotope study. Environ. Sci. Technol. 39, 7576–7584. 10.1021/es0506657 16245830

[B74] Vanitha P. O'Brien P. (2008). Phase Control In The Synthesis Of Magnetic iron sulfide nanocrystals from a cubane-type Fe-S cluster. J. Am. Chem. Soc. 130, 17256–17257. 10.1021/ja8078187 19035629

[B75] Wadia C. Wu Y. Gul S. Volkman S. Guo J. Paul Alivisatos A. (2009). Surfactant-assisted hydrothermal synthesis of single phase pyrite FeS_2_ nanocrystals. Chem. Mater. 21, 2568–2570. 10.1021/cm901273v

[B76] Wang X. Zhou W. Zhou Z. An Y. Wu S. (2013). Shape-controlled synthesis of iron sulfide nanostructures by thermal decomposition of organometallic precursors. Mater. Sci. Semicond. Proc. 16, 530–536. 10.1016/j.mssp.2012.10.002

[B77] Wang Z. Hu T. Liang R. Wei M. (2020). Application of zero-dimensional nanomaterials in biosensing. Front. Chem. 8:320. 10.3389/fchem.2020.00320 32373593 PMC7182656

[B78] Watson J. Cressey B. Roberts A. Ellwood D. C. Charnock J. Soper A. K. (2000). Structural and magnetic studies on heavy-metal-adsorbing iron sulphide nanoparticles produced by sulphate-reducing bacteria. J. Magn. Magn. Mater. 214, 13–30. 10.1016/S0304-8853(00)00025-1

[B79] Watson J. Ellwood D. Deng Q. Mikhalovsky S. Hayter C. E. Evans J. (1995). Heavy metal adsorption on bacterially produced FeS. Minerals Eng. 8, 1097–1108. 10.1016/0892-6875(95)00075-2

[B80] Watson J. Ellwood D. Soper A. Charnock J. (1999). Nanosized strongly-magnetic bacterially-produced iron sulfide materials. J. Magnetism Magnetic Mater. 203, 69–72. 10.1016/S0304-8853(99)00191-2

[B81] Wei H. Wang E. (2013). Nanomaterials with enzyme-like characteristics (nanozymes): next-generation artificial enzymes. Chem. Soc. Rev. 42, 6060–6093. 10.1039/c3cs35486e 23740388

[B82] Xiao S. Cheng M. Zhong H. Liu Z. Liu Y. Yang X. (2020). Iron-mediated activation of persulfate and peroxymonosulfate in both homogeneous and heterogeneous ways: a review. Chem. Eng. J. 384:123265 10.1016/j.cej.2019.123265

[B83] Xie J. Zhang X. Wang H. Zheng H. Huang Y. Xie J. (2012). Analytical and environmental applications of nanoparticles as enzyme mimetics. Trac Trends Analyt. Chemistry 39, 114–129. 10.1016/j.trac.2012.03.021

[B84] Xu Z. Qiu Z. Liu Q. Huang Y. Li D. Shen X.. (2018). Converting organosulfur compounds to inorganic polysulfides against resistant bacterial infections. Nat. Commun. 9:3713. 10.1038/s41467-018-06164-7 30213949 PMC6137151

[B85] Yang K. Yang G. Chen L. Cheng L. Wang L. Ge C.. (2015). FeS nanoplates as a multifunctional nano-theranostic for magnetic resonance imaging guided photothermal therapy. Biomaterials 38, 1–9. 10.1016/j.biomaterials.2014.10.052 25457978

[B86] Yang W. Xiang C. Xu Y. Chen S. Zeng W. Liu K.. (2020). Albumin-constrained large-scale synthesis of renal clearable ferrous sulfide quantum dots for T1-Weighted MR imaging and phototheranostics of tumors. Biomaterials 255:120186. 10.1016/j.biomaterials.2020.120186 32585478

[B87] Yao W. Zhu H. Li W. Yao H. Wu Y. Yu S. (2013). Intrinsic peroxidase catalytic activity of Fe_7_S_8_ nanowires templated from [Fe_16_S_20_]/diethylenetriamine hybrid nanowires. Chem. Plus Chem. 78, 723–727. 10.1002/cplu.201300075 31986636

[B88] Yosida K. (1951). Note on the magnetic properties of the FeS_n_ system. Progr. Theor. Phys. 6, 356–365. 10.1143/ptp/6.3.356

[B89] Zhang L. Webster T. (2009). Nanotechnology and nanomaterials: promises for improved tissue regeneration. Nano Today 4, 66–80. 10.1016/j.nantod.2008.10.014

[B90] Zhang M. Cui Z. Jiang H. (2018). Relative stability of FeS_2_ polymorphs with the random phase approximation approach. J. Mater. Chem. A 6:6606 10.1039/C8TA00759D

[B91] Zhao Q. Yi X. Li M. Zhong X. Shi Q. Yang K. (2016). High near-infrared absorbing Cu_5_FeS_4_ nanoparticles for dual-modal imaging and photothermal therapy. Nanoscale 8, 13368–13376. 10.1039/C6NR04444A 27341480

